# USP7 inhibits Wnt/β-catenin signaling through promoting stabilization of Axin

**DOI:** 10.1038/s41467-019-12143-3

**Published:** 2019-09-13

**Authors:** Lei Ji, Bo Lu, Raffaella Zamponi, Olga Charlat, Robert Aversa, Zinger Yang, Frederic Sigoillot, Xiaoping Zhu, Tiancen Hu, John S. Reece-Hoyes, Carsten Russ, Gregory Michaud, Jan S. Tchorz, Xiaomo Jiang, Feng Cong

**Affiliations:** 1 Novartis Institutes for Biomedical Research, Novartis Pharma AG, Cambridge, MA USA; 2 Novartis Institutes for Biomedical Research, Novartis Pharma AG, Basel, Switzerland; 3 Present Address: Azienda USL—IRCCS di Reggio Emilia, Viale Risorgimento 80, 42123 Reggio Emila, Italy

**Keywords:** Cell signalling, Morphogen signalling, Ubiquitylation, Deubiquitylating enzymes

## Abstract

Axin is a key scaffolding protein responsible for the formation of the β-catenin destruction complex. Stability of Axin protein is regulated by the ubiquitin-proteasome system, and modulation of cellular concentration of Axin protein has a profound effect on Wnt/β-catenin signaling. Although E3s promoting Axin ubiquitination have been identified, the deubiquitinase responsible for Axin deubiquitination and stabilization remains unknown. Here, we identify USP7 as a potent negative regulator of Wnt/β-catenin signaling through CRISPR screens. Genetic ablation or pharmacological inhibition of USP7 robustly increases Wnt/β-catenin signaling in multiple cellular systems. USP7 directly interacts with Axin through its TRAF domain, and promotes deubiquitination and stabilization of Axin. Inhibition of USP7 regulates osteoblast differentiation and adipocyte differentiation through increasing Wnt/β-catenin signaling. Our study reveals a critical mechanism that prevents excessive degradation of Axin and identifies USP7 as a target for sensitizing cells to Wnt/β-catenin signaling.

## Abstract (web summary)

Axin is a scaffolding protein known for its role in Wnt signalling that can be marked with a variety of post-translational modifications. Here, Cong et al. demonstrate that USP7 de-ubiquinates Axin and that canonical Wnt signaling output can be increased with USP7 inhibitors.

## Introduction

The Wnt/β-catenin signaling pathway is an evolutionarily conserved signaling pathway that plays essential roles in embryonic development and adult tissue homeostasis^1–4^. The Wnt/β-catenin pathway regulates proteolysis of transcription cofactor β-catenin. In the absence of Wnt, β-catenin is bound to the β-catenin destruction complex, which contains the scaffolding protein Axin, the tumor suppressor adenomatous polyposis coli (APC), glycogen synthase kinase 3 (GSK3) and casein kinase 1α (CK1α). In this complex, β-catenin is phosphorylated by CK1α and GSK3, and targeted for ubiquitin-dependent proteasomal degradation. In the presence of Wnt, Wnt ligand binds to the Frizzled receptor and the LRP5/6 co-receptor and induces phosphorylation of LRP5/6, which leads to decreased activity of the β-catenin destruction complex and stabilization of β-catenin. Accumulated β-catenin enters the nucleus, binds to TCF family transcription factors, and enhances the transcription of β-catenin target genes. Wnt/β-catenin signaling plays important roles in many important biological processes. For instance, Wnt/β-catenin signaling plays a pivotal role in the mesenchymal stem cell (MSC) lineage specification and early differentiation^5^. Wnt/β-catenin signaling drives osteoblast differentiation of mesenchymal stem cells^6^, while suppresses the adipogenic program^7^. Aberrant Wnt/β-catenin signaling has been associated with many human diseases, such as degenerative diseases and cancer^2^.

As a key scaffolding protein, Axin directly interacts with β-catenin, GSK3, and APC, and is directly responsible for the formation of the β-catenin destruction complex. Since the concentration of Axin is correlated with the concentration of the β-catenin destruction complex, mechanisms that control the protein level of Axin have major impacts on Wnt/β-catenin signaling. Through genetic and chemical genetic screens, we have previously identified two independent E3 ubiquitin systems that promote Axin degradation. Tankyrase binds to the N-terminus of Axin and promotes PARsylation of Axin^8^, and E3 RNF146 mediates PARsylation-dependent ubiquitination and degradation of Axin^9^. Tankyrase inhibitors strongly inhibit Wnt signaling through stabilizing Axin^8,10^. In addition, E3 SIAH binds to the GSK3-binding domain of Axin and mediates Wnt-induced degradation of Axin^11^, which serves as a positive feedback mechanism to amplify Wnt/β-catenin signaling. Although mechanisms that promote Axin degradation and their importance in Wnt/β-catenin signaling are established, the deubiquitinase that removes polyubiquitin chain from Axin to prevent its excessive degradation has been mysterious. Significantly, since Wnt/β-catenin signaling is exquisitely sensitive to the concentration of Axin, small molecules blocking deubiquitination of Axin are expected to sensitize cells to Wnt signaling and might have therapeutic values.

Ubiquitination is an enzymatic process by which proteins are modified with ubiquitin chains and this process can be reversed by deubiquitinases (DUBs). The process of ubiquitination and deubiquitination is extremely dynamic and involves specific protein-protein interactions. USP7 (herpesvirus associated ubiquitin specific protease, HAUSP), is an ubiquitin hydrolyzing enzyme belonging to the ubiquitin specific protease (USP) family. USP7 has a diverse array of substrates, such as p53^12^, MDM2^13,14^, HDMX^14^, N-MYC^15^, PTEN^16^, FOXP3^17^, Gli^18^, Tip60^19^, and ERCC6^20^. Through its deubiquitination activity, USP7 regulates the stability, function, or localization of its substrates, and plays important roles in cancer development, cell signaling, DNA damage repair, epigenetic regulation, and immune responses (reviewed by^21^).

Here, we identify USP7 as a potent negative regulator of Wnt/β-catenin signaling. Mechanistically, USP7 directly interacts with Axin through its N-terminal TRAF domain and stabilizes Axin by erasing the poly-ubiquitination modification. Genetic or pharmacological inhibition of USP7 promotes osteoblast differentiation and attenuates adipocyte differentiation through activation of Wnt signaling. Our work reveals USP7 as a bona fide deubiquitinase for Axin and demonstrates its negative role in Wnt/β-catenin signal transduction.

## Results

### USP7 is a negative regulator of Wnt/β-catenin signaling

**Fig. 1:**
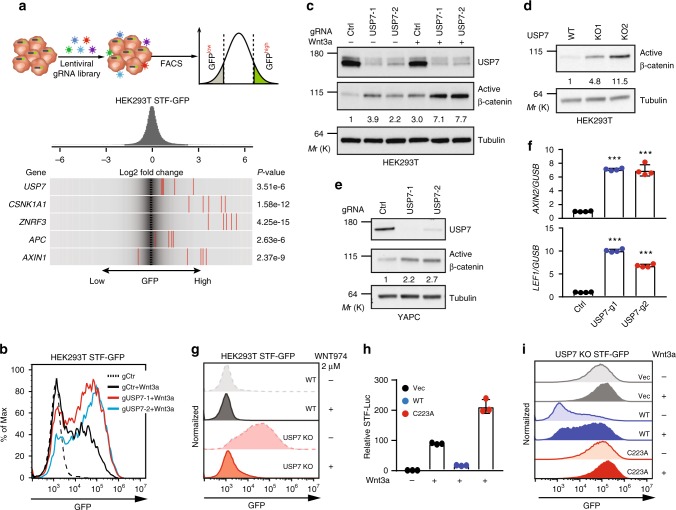
USP7 is a potent negative regulator of Wnt/β-catenin signaling. **a** Top panel, infographic description of pooled CRISPR screen. Bottom panel, frequency histograms of gRNAs identified in the high GFP population of HEK293T pooled CRISPR screen. The RSA *P*-value is shown along with the log2 fold change of the five gRNAs per gene for the indicated set of genes. gRNAs targeting indicated genes are shown by the red lines. **b** Knockout of USP7 by independent gRNAs enhances Wnt3a-induced STF-GFP in FACS assay. STF-GFP reporter was induced by 10% Wnt3a conditioned medium (Wnt3a CM) overnight. The FACS data is a representative from three independent experiments. **c** Knockout of USP7 in HEK293T cells enhances Wnt3a-induced accumulation of active β-catenin. Cells were treated with control medium or 10% Wnt3a CM overnight. The intensity of active β-catenin and Tubulin bands was quantified by ImageJ, and quantification of normalized active β-catenin band intensity is shown. **d** HEK293T USP7 knockout clones express higher levels of active β-catenin as compared to wild-type cells in the absence of exogenous Wnt. **e**, **f** Knockout of USP7 in YAPC cells increases the protein level of active β-catenin (**e**) and mRNA level of β-catenin target genes. Error bars denote the SD between four replicates; one-way ANOVA was used to determine the statistical significance; ***p value ≤ 0.001 (**f**). **g** Porcupine inhibitor (WNT974) blocks the high expression of STF-GFP in HEK293T USP7 knockout cells. Wild-type and USP7 knockout cells were incubated with DMSO or 2 µM WNT974 for five days, and STF-GFP was determined by FACS analysis. The FACS data is a representative from three independent experiments. **h** Overexpression of wild-type (WT) USP7, but not the C223A mutant, represses Wnt3a-induced STF reporter in HEK293T cells. Error bars denote the SD between three replicates. **i** Ectopic expression of wild-type USP7, but not empty vector (Vec) or the C223A mutant, suppresses the high level of STF-GFP in USP7 KO cells. Source data for Fig. 1c, d, e, f, and h are provided as Source Data file

In order to discover unknown regulators of Wnt/β-catenin signaling, we performed genome-wide CRISPR screens in HEK293T cells stably expressing Cas9 and SuperTopFlash-GFP (STF-GFP), a transcription reporter with multiple TCF binding sites. STF-GFP reporter cells were mutagenized by a pooled lenti-viral guide RNA (gRNA) library, treated with Wnt3a conditioned medium (CM) and subjected to FACS analysis. Cells expressing low GFP signal and high GFP signal were collected and subjected to next generation sequencing (NGS) and informatics analysis (Fig. 1a, top panel). Guides overrepresented in the GFP-high population are expected to enhance STF reporter activity. Known negative regulators (*CSNK1A1*, *ZNRF3*, *APC*, and *AXIN1*) of Wnt signaling scored clearly in this screen (Fig. 1a, bottom panel), validating the screening strategy. Surprisingly, USP7, a proposed positive regulator of Wnt/β-catenin signaling^22–24^, scored as a negative regulator of Wnt signaling in the screen (Fig. 1a, bottom panel and Supplementary Dataset 1).

To validate screening results, independent USP7 gRNAs were introduced into HEK293T cells by lenti-viral transduction, and the pool of USP7 knockout cells were treated with Wnt3a CM and subjected to FACS analysis. As seen in Fig. 1b, USP7 knockout significantly enhanced Wnt3a-induced STF-GFP in HEK293T cells. Consistently, knockout of USP7 enhanced Wnt3a-induced accumulation of active β-catenin in HEK293T cells (Fig. 1c). We also generated two independent HEK293T USP7 knockout (KO) clones using CRISPR (Supplementary Fig. 1a and b). Compared with control cells, both HEK293T USP7 knockout clones expressed higher level of active β-catenin in the absence of exogenous Wnt treatment (Fig. 1d). We next examined the effect of USP7 knockout in other cell lines. Pool of YAPC USP7 knockout cells expressed higher level of active β-catenin (Fig. 1e) and β-catenin target genes (Fig. 1f) in the absence of exogenous Wnt. Knockout of USP7 also increased expression of active β-catenin and β-catenin target gene in Huh7 (Supplementary Fig. 1c), another cell line with autocrine Wnt signaling. Consistently, HEK293T and YAPC USP7 knockout cells expressed higher level of STF-GFP as compared to control cells (Fig. 1g and Supplementary Fig. 1d). Treatment of porcupine inhibitor WNT974^25^ completely suppressed high expression of STF-GFP and active β-catenin in HEK293T and YAPC USP7 knockout cells (Fig. 1g and Supplementary Fig. 1d–f), suggesting that USP7 deficiency sensitizes cells to β-catenin signaling mediated by endogenous Wnt proteins.

We next sought to determine whether overexpression of USP7 affects Wnt/β-catenin signaling. HEK293T STF-luciferase (STF-Luc) cells stably expressing empty vector, wild-type USP7 (WT), or the USP7 catalytic dead mutant C223A^26^ were generated and treated with Wnt3a CM. Ectopic expression of wild-type USP7 strongly suppressed Wnt3a-induced STF reporter and β-catenin accumulation (Fig. 1h and Supplementary Fig. 1g). In contrast, ectopic expression of USP7 C223A mutant slightly increased Wnt3a-induced STF reporter (Fig. 1h), presumably through its dominant negative function against endogenous USP7. In addition, we performed the rescue experiment by reintroducing wild-type USP7 and the C223A mutant into USP7 knockout HEK293T STF-GFP cells. USP7 knockout cells expressed a high level of STF-GFP, which was strongly suppressed by wild-type USP7, but not the C223A mutant (Fig. 1i and Supplementary Fig. 1h). Combining loss-of-function, gain-of-function and cDNA rescue data, these results strongly suggest that USP7 negatively regulates Wnt/β-catenin signaling and this function is dependent on its deubiquitinase activity.

### USP7 inhibitors augment Wnt/β-catenin signaling

**Fig. 2:**
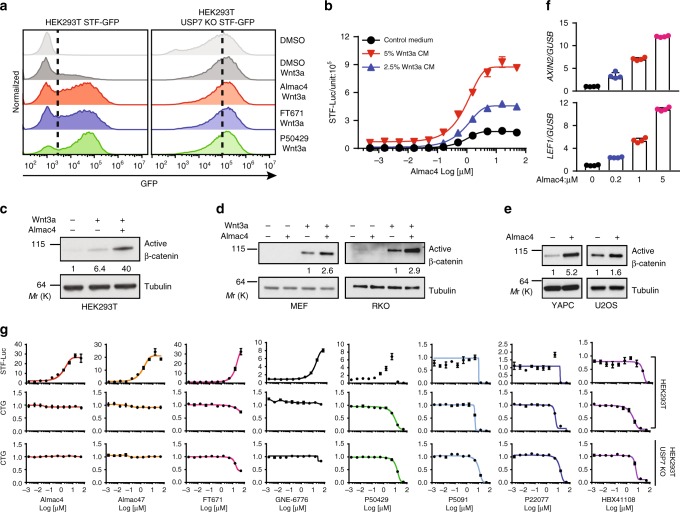
USP7 inhibitors augment Wnt/β-catenin signaling. **a** USP7 inhibitors potentiate Wnt3a-induced STF-GFP in HEK293T cells, but not in HEK293T USP7 KO cells, as determined by FACS assay. Wild-type and USP7 knockout STF-GFP reporter cells were pretreated with DMSO, 1 µM Almac4, 5 µM FT671 or 10 µM P504290 for 24 h and stimulated with 10% Wnt3a CM overnight. **b** Almac4 increases STF-Luc activity in a dose-dependent manner in the presence or absence of exogenous Wnt3a in HEK293T cells. Error bars denote the SD between four replicates. **c**–**e** Almac4 enhances accumulation of active β-catenin. HEK293T, RKO and MEF cells were pretreated with DMSO or Almac4 (1 µM for HEK293T and 2 µM for RKO and MEF) for 24 h, and followed by overnight Wnt3a CM stimulation. YAPC and U2OS cells were treated with DMSO or 2 µM Almac4 for 2 days. **f** Almac4 increases the expression of β-catenin target genes in a dose-dependent manner in YAPC cells. YAPC cells were treated with Almac4 at indicated doses for 2 days and total RNA was prepared for RT-PCR assay. Error bars denote the SD between four replicates. **g** HEK293T (top and middle panel) and HEK293T USP7 KO (bottom panel) cells were pretreated with increasing doses of USP7 inhibitors for 24 h, and stimulated with 5% Wnt3a CM overnight. STF-Luc reporter activity was measure by Bright-Glo assay and viability of cells was assessed using Cell Titer-Glo (CTG) assay. Source data for Fig. 2b, c, d, e, f, and g are provided as Source Data file

After establishing a critical role of USP7 in controlling Wnt/β-catenin signaling, we sought to use USP7 inhibitors to further study this regulation. MDM2 is a well-established substrate of USP7. Inhibition of USP7 leads to proteasomal degradation of MDM2 and stabilization of p53, resulting in cell cycle arrest and apoptosis of cancer cells^13,27,28^. Because of this important function of USP7, significant efforts have been made to develop small molecule USP7 inhibitors to treat cancers with wild-type p53. However, early USP7 inhibitors have low potency/selectivity^29^. Although these inhibitors stabilize p53 protein, it is still not clear whether their cellular toxicity is solely mediated by USP7 inhibition. Recently, USP7 inhibitors with significantly improved potency and selectivity have been reported. We found that the new generation of USP7 inhibitors Almac4^30^, FT671^29^, and P50429^31^ strongly increased STF-GFP in parental HEK293T cells, but not in HEK293T USP7 knockout cells (Fig. 2a). Almac4 potentiated STF-Luc activity in HEK293T cells in a dose-dependent manner with or without exogenous Wnt3a (Fig. 2b). Almac4 also enhanced Wnt-induced accumulation of active β-catenin in various cell lines including HEK293T, MEF, RKO, YAPC, and U2OS (Fig. 2c–e). Consistently, Almac4 increased the expression of β-catenin target genes *AXIN2* and *LEF1* in YAPC cells (Fig. 2f).

Interestingly, USP7 has been reported as a positive regulator of Wnt/β-catenin signaling as early generation of USP7 inhibitors can inhibit Wnt/β-catenin signaling^23,24^. However, interpretation of such data is complicated by low potency and strong off-target activity of these early compounds. We measured effects of various USP7 inhibitors on Wnt/β-catenin signaling and cell proliferation using STF-Luc assay and Cell Titer-Glo (CTG) assay in a dose response manner (Fig. 2g). All new generation USP7 inhibitors, including Almac4^30^, Almac47^32^, P50429^31^, FT671^29^, and GNE-6776^33^, increased STF reporter. Early generation of USP7 inhibitors P5091^34^, P22077^35^, and HBX41108^36^ decreased STF reporter, as reported earlier^23,24^. However, early generation of USP7 inhibitors strongly inhibited CTG in parental HEK293T cells and USP7 KO cells at the same concentrations as they inhibited STF reporter, suggesting that these compounds have clear off-target activities and their inhibitory effect on Wnt/β-catenin signaling is likely off-target. Of all compounds tested, Almac4 and Almac47 have the strongest Wnt stimulatory activities. Both compounds also do not affect proliferation of HEK293T USP7 KO cells at all concentrations tested, suggesting that Almac4 and Almac47 have the least off-target activities. Together these data suggest that pharmacological inhibition of USP7 enhances Wnt/β-catenin signaling in different cell lines.

### USP7 promotes deubiquitination and stabilization of Axin

Axin is a key scaffolding protein of the β-catenin destruction complex, and modulation of the concentration of Axin has a profound effect on Wnt/β-catenin signaling. Although the E3s responsible for Axin ubiquitination have been discovered, the DUB that protects ubiquitinated Axin from degradation remains unknown. Since the catalytic activity of USP7 is crucial for its negative role in Wnt/β-catenin signaling, we asked whether USP7 is a bona fide DUB for Axin.

**Fig. 3:**
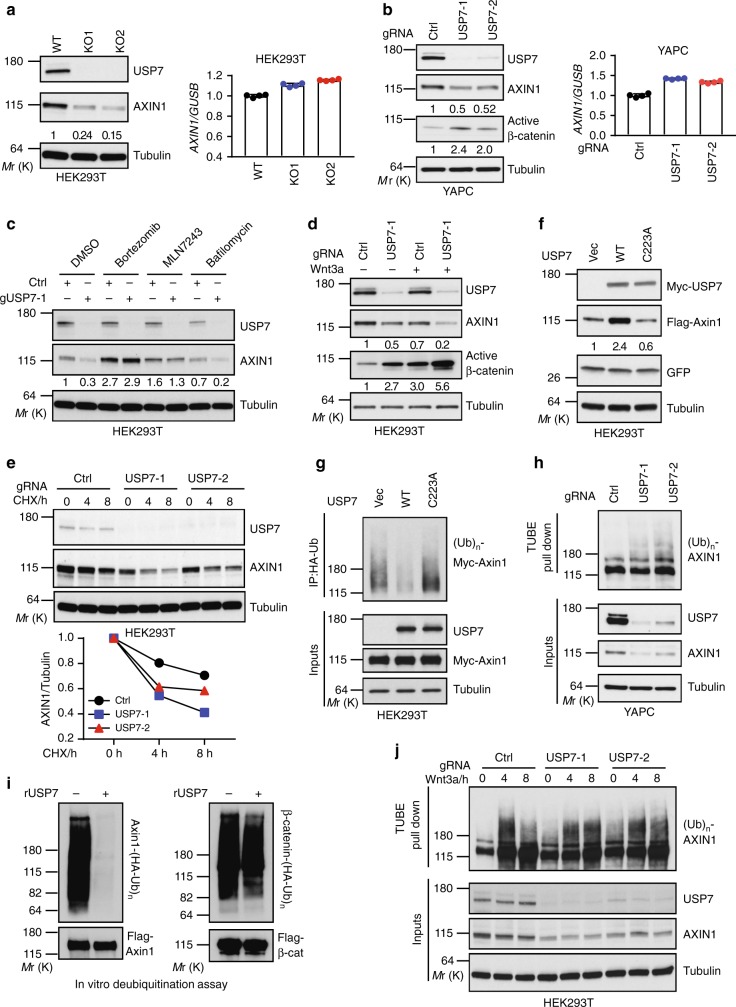
USP7 promotes deubiquitination and stabilization of Axin. **a**, **b** Knockout of USP7 decreases the protein level, but not the mRNA level, of AXIN1 in HEK293T cells (**a**) and YAPC cells (**b**). Error bars denote the SD between four replicates. **c** Proteasome inhibitor Bortezomib and E1 inhibitor MLN7243, but not lysosome inhibitor Bafilomycin A1, inhibit the effect of USP7 knockout on the expression of AXIN1 protein in HEK293T cells. HEK293T control and USP7 gRNA bearing cells were treated with DMSO, 5 µM Bortezomib, 5 µM MLN7243 or 0.1 µM Bafilomycin A1 overnight. **d** Knockout of USP7 promotes Wnt3a-induced AXIN1 degradation and accumulation of active β-catenin in HEK293T cells. HEK293T control and USP7 gRNA bearing cells were incubated with control medium or 20% Wnt3a CM overnight. **e** Knockout of USP7 decreases the protein stability of AXIN1 upon Wnt3a treatment in HEK293T cells. Cells were treated with 100 µg/ml cycloheximide (CHX) to block de novo protein synthesis and simultaneously stimulated with 20% Wnt3a CM to induce AXIN1 degradation. The intensity of AXIN1 and Tubulin bands was quantified by ImageJ, and quantification of normalized AXIN1 band intensity is shown in the bottom panel. **f** Overexpression of wild-type USP7, but not the C223A mutant, stabilizes co-expressed Axin1 protein. GFP serves as a control for transfection efficiency. **g** Overexpression of wild-type USP7, but not the C233A mutant, decreases poly-ubiquitination of co-expressed Axin1. **h** Knockout of USP7 enhances poly-ubiquitination of endogenous AXIN1 in YAPC cells. Control and USP7 gRNA bearing YAPC cells were incubated with 10 µM MG132 overnight and harvested for TUBE assay. **i** Recombinant USP7 protein (rUSP7) efficiently erases poly-ubiquitination of Axin1 but not β-catenin in an in vitro deubiquitination assay. **j** Wnt3a-induced poly-ubiquitination of endogenous AXIN1 is sustained upon USP7 knockout in HEK293T cells. HEK293T control and USP7 gRNA bearing cells were treated with 30% Wnt3a CM and 20 µM MG132 and harvested at different time points for TUBE assay., Source data for Fig. 3a, b, c, d, e, f, g, h, i, and j are provided as Source Data file

To address this question, we first examined the expression of key components of the β-catenin destruction complex in HEK293T Cas9 cells expressing control or USP7 gRNA. In the absence of exogenous Wnt, knockout of USP7 decreased the expression of AXIN1, but not other components of the β-catenin destruction complex (APC, GSK3β and CK1) (Supplementary Fig. 3a). In HEK293T USP7 KO clones, the protein level, but not mRNA level, of AXIN1 was much lower than that of control cells in the absence of exogenous Wnt (Fig. 3a). Similarly, knockout of USP7 decreased the protein level, but not mRNA level, of AXIN1 and increased active β-catenin in YAPC cells (Fig. 3b). Decreased expression of AXIN1 protein in HEK293T USP7 KO cells was rescued by proteasome inhibitor Bortezomib and E1 inhibitor MLN7243, but not lysosome inhibitor Bafilomycin A1 (Fig. 3c). We next examined the effect of USP7 knockout on Wnt-induced Axin degradation. In agreement with previous observations^11,37–39^, Wnt3a increased the degradation of AXIN1, and this effect was further enhanced by USP7 knockout (Fig. 3d). Pharmacological inhibition of USP7 using Almac4 decreased expression of AXIN1 and increased accumulation of β-catenin with or without exogenous Wnt (Supplementary Fig. 2a–e). When the de novo protein synthesis was blocked by cycloheximide (CHX), USP7 knockout shortened the half-life of AXIN1 protein upon Wnt3a stimulation (Fig. 3e and Supplementary Fig. 3b). Consistent with loss-of-function data, overexpression of wild-type USP7, but not the catalytic dead mutant (C223A), increased the protein level of coexpressed Axin1 protein in HEK293T cells (Fig. 3f). Taken together, these data strongly suggest that USP7 stabilizes Axin protein by inhibiting its proteasomal degradation.

Since USP7 stabilizes its substrates through promoting their deubiquitination, we examined whether USP7 could affect ubiquitination of Axin. Overexpression of wild-type USP7, but not the C233A mutant, decreased poly-ubiquitination of ectopically expressed Axin1 (Fig. 3g). Consistently, knockout of USP7 in YAPC cells enhanced poly-ubiquitination of endogenous AXIN1 in the TUBE assay (Fig. 3h). We next tested whether Axin is a direct substrate of USP7 through in vitro deubiquitination assay. Since USP7 has recently been suggested to deubiquitinate and stabilize β-catenin^24^, we included β-catenin as a control. Cells coexpressing HA-Ub and Flag-tagged Axin1 or β-catenin were treated with MG132. Polyubiquitinated Axin1 or β-catenin was purified using Flag antibody-conjugated beads under denaturing condition, and subjected to an in vitro deubiquitination assay by co-incubation with recombinant full-length USP7 (Supplementary Fig. 3c). Consistent with our cellular data, recombinant USP7 completely erased poly-ubiquitination modification of Axin1 (Fig. 3i, left panel). In contrast, recombinant USP7 only had a minor effect on poly-ubiquitination of β-catenin in the same assay condition (Fig. 3i, right panel). These results suggest that polyubiquitinated Axin1, but not β-catenin, is an efficient substrate of USP7.

Since USP7 knockout promotes Wnt-induced Axin degradation (Fig. 3d, e), we next examined whether USP7 knockout affects Wnt-induced Axin ubiquitination. In control HEK293T cells, poly-ubiquitination of AXIN1 was induced by Wnt3a CM treatment at 4 h time point, while it decreased at 8 h time point (Fig. 3j). In contrast, sustained poly-ubiquitination of AXIN1 was seen at 4 h and 8 h time points in USP7 knockout cells (Fig. 3j), suggesting USP7 counters Wnt-induced ubiquitination of Axin.

We have previously demonstrated that both RNF146 and SIAH1 promote the poly-ubiquitination and degradation of Axin. Coexpression of USP7 suppressed either RNF146 or SIAH1-induced poly-ubiquitination of Axin (Supplementary Fig. 3d). Inhibition of SIAH1 and TNKS, either individually or in combination, reduced STF-GFP in USP7 null HEK293T cells (Supplementary Fig. 3e). Alamc4-enhanced AXIN1 degradation was blocked in SIAH1 and RNF146 deficient HEK293T cells (Supplementary Fig. 3f). In addition, USP7 inhibition-induced accumulation of active β-catenin was abolished in HEK293T AXIN1/AXIN2 double knockout cells (Supplementary Fig. 3g). Taken together, these results suggest that USP7 inhibits Wnt signaling through counteracting RNF146 and SIAH1-induced ubiquitination and degradation of Axin.

### TRAF domain in USP7 binds to Axin

**Fig. 4:**
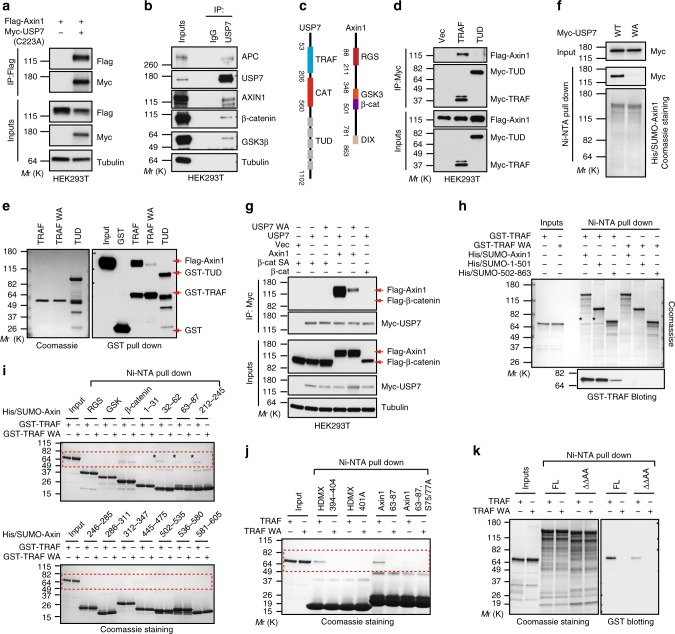
USP7 directly binds to Axin through its TRAF domain. **a** Ectopically expressed Axin1 and USP7 C223A mutant interact with each other in co-immunoprecipitation assay. **b** Endogenous AXIN1 and other major components of β-catenin destruction complex are co-immunoprecipitated with endogenous USP7 in the co-immunoprecipitation assay. **c** Schematic diagrams of Axin1 and USP7 proteins. **d** Ectopically expressed TRAF domain, but not TUD domain, of USP7 interacts with Flag-Axin1 in co-immunoprecipitation assay. **e** Purified recombinant GST-TRAF, but not GST-TRAF W165A mutant or GST-TUD, interacts with ectopically expressed Flag-Axin1 in GST pulldown assay. Quality of GST fusion proteins was determined by coomassie staining (left panel) and pulled down proteins were revealed by immunoblotting with Flag and GST antibodies (right panel). **f** Purified recombinant His-SUMO-Axin1 selectively interacts with ectopically expressed wild-type Myc-USP7, but not Myc-USP7 W165A mutant, in Ni-NTA pulldown assay. **g** Interaction between ectopically expressed Flag-Axin1 or Flag-β-catenin and Myc-USP7 in co-immunoprecipitation assay. **h** Purified His-SUMO tagged full-length and the N-terminal fragment (a.a.1–501) of Axin1 exhibit similar binding to purified GST-TRAF in Ni-NTA pulldown assay. Pulled down GST-TRAF proteins are denoted by asterisks. **i** Interaction between small fragments of Axin1 and USP7-TRAF domain in Ni-NTA pulldown assay. Three small fragments of Axin1 selectively interacting with GST-TRAF are denoted by asterisks. **j** S75/77 A mutation attenuates the interaction between purified His-SUMO-Axin1 a.a. 63–87 and GST-TRAF protein in the Ni-NTA pulldown assay. His-SUMO-HDMX a.a. 394–404 and its S401A mutant serve as positive and negative control respectively. **k** Axin1 mutant (ΔΔAA) with deletion of a.a. 32–62 and a.a. 212–245 and mutation of S75/77 shows reduced interaction with GST-TRAF as compared with full length Axin1 (FL). Protein quality was determined by coomassie staining, and pulled down GST-TRAF was revealed by immunoblotting with anti-GST antibody. Source data for Fig. 4a, b, d, e, f, g, h, i, j and k are provided as Source Data file

Based on the functional data discussed above, we speculated that USP7 might physically interact with Axin to promote its deubiquitination. Indeed, ectopically expressed Flag-Axin1 and Myc-USP7 or its catalytic mutant bound to each other in co-immunoprecipitation assay (Fig. 4a and Supplementary Fig. 4a). Further, interaction between endogenous USP7 and AXIN1 and other components of β-catenin destruction complex was detected in HEK293T cells (Fig. 4b), suggesting that USP7 is associated with the destruction complex. Consistent with these results, USP7 was a hit in Axin1 tandem affinity protein purification mass spectrometry screen^40^.

We next examined which domain of USP7 is responsible for Axin interaction. USP7 contains three major domains, including an amino-terminal tumor necrosis associated factor-like (TRAF) domain, a middle catalytic domain and a carboxyl-terminal tandem UBL domain (TUD), which contains five ubiquitin-like folds^41^ (Fig. 4c). Both the TRAF domain and the TUD domain of USP7 can directly mediate substrate recognition^42^. We tested the interaction between Flag-tagged Axin1 and Myc-tagged TRAF domain or TUD domain of USP7 in co-immunoprecipitation assay. As seen in Fig. 4d, Axin1 was co-immunoprecipitated with the TRAF domain, but not the TUD domain, of USP7. In agreement with the co-immunoprecipitation data, purified GST-TRAF, but not GST-TUD, was able to pull down Flag-Axin1 from cell lysates in the GST pulldown assay (Fig. 4e). It has been shown previously that W165 of the TRAF domain of USP7 plays a major role in substrate recognition and W165A mutation of TRAF domain strongly decreases the interaction between USP7 and many of its substrates^43–45^. Behaving similarly as established USP7 substrates, Axin1 showed strongly reduced interaction with GST-TRAF W165A (WA) mutant (Fig. 4e). In a reciprocal pulldown assay using cell lysates containing ectopically expressed USP7, purified His/SUMO-Axin1 proteins selectively pulled down wild-type USP7 but not the WA mutant (Fig. 4f). In addition, AXIN2 also interacted with wild-type USP7 but not the WA mutant in co-immunoprecipitation assay (Supplementary Fig. 4b). These results suggest that the TRAF domain of USP7 interacts with AXIN1 and AXIN2 in a similar manner as it binds to other USP7 substrates.

Direct binding between USP7 and β-catenin has recently been suggested^24^. To rule out the possibility that the interaction between USP7 and Axin is mediated by β-catenin, we included β-catenin as a control in USP7 co-immunoprecipitation assay. Consistent with the pull down data, Flag-Axin1strongly interacted with ectopically expressed wild-type USP7, but not the WA mutant (Fig. 4g). Note that coexpression of USP7 did not affect Axin1 expression as higher ratio of Axin1 to USP7 expression plasmid was used in this experiment. Interestingly, we could not detect a clear interaction between USP7 and either wild-type β-catenin or β-catenin S37A mutant in this experimental setting (Fig. 4g and Supplementary Fig. 4c). Lack of a clear interaction between USP7 and β-catenin is consistent with our earlier finding that polyubiquitinated β-catenin serves as a poor substrate of USP7 (Fig. 3i).

We next examined which region of Axin1 is responsible for USP7 interaction. Axin contains four major domains, RGS domain, GSK3-binding domain, β-catenin binding domain and DIX domain (Fig. 4c). We first tested whether any of these domains is required for USP7 binding. As seen in Supplementary Fig. 4d, full-length Axin1 and Axin1 mutants with these domains individually deleted bound to USP7 TRAF domain equally well in the GST pulldown assay, suggesting that these domains are not required for Axin binding. We next purified His-SUMO tagged full-length Axin1 (a.a. 1–863), Axin1 a.a. 1–501, and Axin1 a.a. 502–863 proteins, incubated them with purified GST-TRAF or GST-TRAF WA mutant, and performed pulldown assay using Ni-NTA resins. As seen in Fig. 4h, full length Axin1 and Axin1 a.a. 1–501 showed similar binding to GST-TRAF, while Axin1 a.a. 502–863 showed much weaker binding. As expected, GST-TRAF WA mutant was not pulled down by Axin1. These results suggest that the amino-terminal two-thirds of AXIN1 is mainly responsible for the direct interaction with USP7. Since USP7 has been shown to bind to linear peptides^43,46,47^, we purified multiple His-SUMO-tagged Axin fragments covering Axin1 a.a. 1–501 and tested them in pulldown assay. As seen in Fig. 4i, three small Axin1 fragments (a.a. 32–62, a.a. 63–87, and a.a. 212–245) bound to GST-TRAF, but not GST-TRAF WA mutant. β-catenin binding domain of Axin bound to both GST-TRAF and GST-TRAF WA mutant (Fig. 4i), but the significance of this binding is not clear since W165A mutation largely abolished the interaction between Axin and USP7 (Fig. 4e–h). The Ser residue of many USP7 binding sites plays a critical role in mediating interaction with the TRAF domain of USP7. Consistent with the previous work, the interaction between HDMX a.a. 394–404 peptide and USP7 TRAF domain was largely abolished by S401A mutation in HDMX (Fig. 4j and Supplementary Fig. 4e). Axin1 a.a. 32–62 and a.a. 212–245 have multiple Ser residues (Supplementary Fig. 4f). There are three Ser residues in a.a. 63–87, and two of them (S75 and S77) are well conserved. In particular, a.a 63–87 contains a ^72^PEGS^75^ motif (Supplementary Fig. 4g) that corresponds to the consensus USP7 TRAF domain binding sequence (P/AxxS)^43^. Notably, mutation of S75 and S77 of Axin1 a.a. 63–87 weakened its interaction with GST-TRAF (Fig. 4j and Supplementary Fig. 4e). In addition, Axin1 mutant with a.a.32–62 and a.a. 212–245 deletion and S75/77A mutation showed decreased interaction with USP7 TRAF domain as compared with wild-type Axin1 (Fig. 4k). These results suggest that USP7 directly interacts with several sites of Axin1 through its TRAF domain.

### USP7 inhibits Wnt-induced osteoblast differentiation

**Fig. 5:**
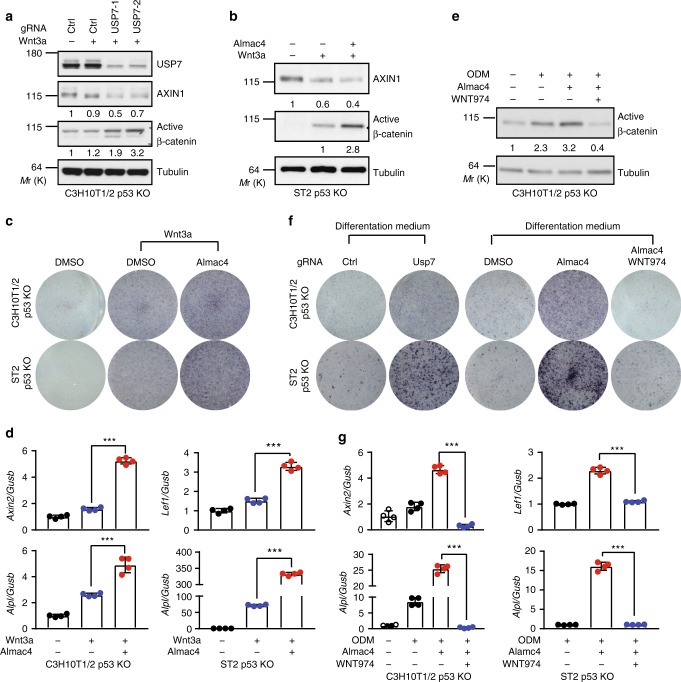
USP7 inhibits Wnt-induced osteoblast differentiation. **a** Knockout of Usp7 enhances Wnt3a-induced active β-catenin accumulation in C3H10T1/2 p53 KO cells. Cells bearing control or Usp7 gRNA were incubated with 10% Wnt3a CM overnight. **b** USP7 inhibitor Almac4 enhances Wnt3a-induced active β-catenin accumulation in ST2 p53 KO cells. Cells were pretreated with DMSO or 2 µM Almac4 for 24hrs, and then incubated with 5% Wnt3a CM overnight. (**c**, **d**) Almac4 promotes Wn3a-induced Alkaline phosphatase staining and β-catenin target gene and Alkaline phosphatase (*Alpl*) expression in C3H10T1/2 p53 KO and ST2 p53 KO cells. Cells were incubated with DMSO, DMSO and 2% Wnt3a CM, or 1 µM Almac4 and 2% Wnt3a CM for seven days and subjected to alkaline phosphatase (ALP) staining (**c**) or RT-PCR assay (**d**). **e** Almac4 enhances osteoblast differentiation medium (ODM)-induced accumulation of active β-catenin. C3H10T1/2 p53 KO cells were cultured in regular medium or differentiation medium in the presence of DMSO, 1 µM Almac4, or 1 µM Almac4 and 2 µM WNT974 for 12 days. **f** Genetic knockout (left panel) or pharmacological inhibition (right panel) of USP7 enhances osteoblast differentiation medium-induced alkaline phosphatase staining in C3H10T1/2 p53 KO and ST2 p53 KO cells, which is abolished by WNT974. Both control and Usp7 gRNA bearing cells were treated with differentiation medium for 12 days before the ALP staining. Conditions are the same as described in Fig. 5e. **g** Almac4 increases osteoblast differentiation medium-induced expression of Alkaline phosphatase (*Alpl*) and β-catenin target gene in C3H10T1/2 p53 KO and ST2 p53 KO cells, which is suppressed by WNT974. In Fig. 5d, g, error bars denote the SD between four replicates; one-way ANOVA was used to determine the statistical significance; ***p value ≤ 0.001. Source data for Fig. 5a, b, d, e, and g are provided as Source Data file

It is well established that Wnt/β-catenin signaling promotes osteoblast differentiation of MSCs^5,6^. Since USP7 negatively regulates Wnt signaling, we studied the function of USP7 in osteoblast differentiation in mouse multipotent mesenchymal stem cells C3H10T1/2 and mouse bone marrow-derived stroma cells ST2. It has been shown previously that ablation of USP7 could increase p53 abundance, which can trigger cell cycle arrest and apoptosis^13^. To avoid complications associated USP7 inhibition-induced p53 accumulation, we knocked out p53 in both C3H10T1/2 and ST2 cells using CRISPR. Upon Wnt3a treatment, knockout of Usp7 increased Wnt3a-induced accumulation of active β-catenin in C3H10T1/2 cells (Fig. 5a). Further, treatment of USP7 inhibitor Almac4 increased accumulation of active β-catenin with or without exogenous Wnt (Fig. 5b and Supplementary Fig. 5a). These results suggest that USP7 negatively inhibits Wnt/β-catenin signaling in C3H10T1/2 and ST2 cells as shown previously in other cell lines.

We next examined whether USP7 inhibition affects Wnt-induced osteoblast differentiation. Alkaline phosphatase is an early marker of osteoblast differentiation. Treatment of Almac4 enhanced Wn3a-induced alkaline phosphatase staining in both C3H10T1/2 and ST2 cells (Fig. 5c). Consistently, Almac4 increased Wnt3a-induced mRNA levels of Alkaline phosphatase (*Alpl*) and β-catenin target gene *Axin2* and *Lef1* in C3H10T1/2 and ST2 cells (Fig. 5d). Axin2 mRNA level in ST2 cells is below the detection limit.

We next tested whether inhibition of USP7 regulates Wnt/β-catenin signaling and osteoblast differentiation in the presence of osteoblast differentiation medium (ODM). ODM increased the level of active β-catenin in C3H10T1/2 cells, which is further enhanced by USP7 inhibitor Almac4, and this effect is abolished by porcupine inhibitor WNT974 (Fig. 5e). In the presence of ODM, genetic knockout or pharmacological inhibition of USP7 in C3H10T1/2 and ST2 cells increased the alkaline phosphatase staining (Fig. 5f) and mRNA expression of *Alpl* and β-catenin target genes (Fig. 5g), which were blocked by WNT974 (Fig. 5f, g). Taken together, these results suggest that USP7 inhibits osteoblast differentiation through attenuating Wnt/β-catenin signaling.

### USP7 promotes adipocyte differentiation through repressing Wnt signaling

**Fig. 6:**
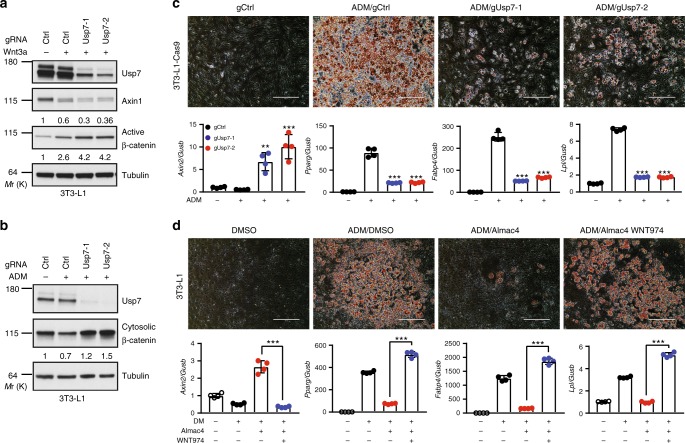
USP7 promotes adipocyte differentiation through inhibiting Wnt signaling. **a** Knockout of Usp7 enhances Wnt3a-induced active β-catenin accumulation and Axin1 degradation in 3T3-L1 cells. Cells were incubated with 10% Wnt3a CM overnight and harvested for immunoblotting. **b** Knockout of Usp7 overcomes adipocyte differentiation medium (ADM)-suppressed expression of cytosolic β-catenin in 3T3-L1 cells. Cells were cultured in regular medium or differentiation medium for four days and harvested for immunoblotting. **c** Knockout of Usp7 attenuates adipocyte differentiation medium-induced lipid droplet formation (top panel), enhances the expression of β-catenin target gene, and suppresses the expression of adipogenic markers (bottom panel) in 3T3-L1 cells. 3T3-L1 cells were subjected to the standard differentiation protocol for ten days, and stained with Oil-Red O (top panels) or subjected to RT-PCR assay (bottom panels). Scale bars, 400 µm. **d** Almac4 blocks adipocyte differentiation medium-induced lipid droplet formation and adipogenic marker expression, which is reversed by porcupine inhibitor WNT974. 3T3-L1 cells were subjected to the standard differentiation protocol in the presence of DMSO, 0.5 µM Almac4, or 0.5 µM Almac4 and 2 µM WNT974 for ten days, and then cells were stained with Oil-Red O (top panels) or subjected to RT-PCR assay (bottom panels). Scale bars, 400 µm. In Fig. 6c, d, error bars denote the SD between four replicates; one-way ANOVA was used to determine the statistical significance; ***p value ≤ 0.001. Source data for Fig. 6a, b, c, and d are provided as Source Data file

It is well known that Wnt/β-catenin signaling inhibits adipocyte differentiation^7,48^. We investigated the role of USP7 in adipocyte differentiation using mouse preadipocyte cell line 3T3-L1^49^. Unlike C3H10T1/2 and ST2, proliferation of 3T3-L1 is not affected by USP7 inhibition although 3T3-L1 expresses wild-type p53^50^. Knockout Usp7 using independent gRNAs enhanced Wnt3a-induced accumulation of active β-catenin (Fig. 6a). Treatment of USP7 inhibitor Almac4 also increased accumulation of active β-catenin (Supplementary Fig. 5b). These results suggest that USP7 inhibits Wnt/β-catenin signaling in 3T3-L1 cells. We next examined the role of USP7 in β-catenin signaling in 3T3-L1 cells treated with adipocyte differentiation medium. In agreement with previous findings^7^, adipocyte differentiation medium decreased the level of cytosolic β-catenin in 3T3-L1 cells (Fig. 6b). Notably, Usp7 knockout significantly increased the level of cytosolic β-catenin (Fig. 6b) and the expression of β-catenin target gene *Axin2* in the presence of adipocyte differentiation medium (Fig. 6c, lower panel). These results suggest that USP7 knockout increased Wnt/β-catenin signaling in cells treated with adipocyte differentiation medium. Consistent with the notion that Wnt/β-catenin signaling blocks adipocyte differentiation, Usp7 knockout strongly inhibited adipocyte differentiation medium-induced lipid droplet formation (Fig. 6c, upper panel) and expression of adipogenic markers such as *Pparg*, *Fabp4*, and *Lpl* (Fig. 6c, lower panel).

It has been suggested that USP7-mediated deubiquitination of the histone acetyltransferase TIP60 promotes adipocyte differentiation^51^. To gain mechanistic insights into regulation of adipogenesis by USP7 in 3T3-L1 cells, we tested whether porcupine inhibitor can overcome the inhibitory effect of USP7 inhibitor on adipocyte differentiation. Significantly, porcupine inhibitor WNT974 completely reversed the USP7 inhibitor-blocked the adipocyte differentiation, as indicated by lipid droplet formation (Fig. 6d, upper panel) and expression of adipogenic markers and β-catenin target gene (Fig. 6d, lower panel). These results suggest that USP7 inhibition blocks adipocyte differentiation mainly through activation of Wnt signaling.

## Discussion

In this study, we have identified USP7 as a potent negative regulator of Wnt signaling through CRISPR screen. USP7 physically interacts with Axin through its TRAF domain and serves as a bona fide deubiquitinase for Axin. Through removing poly-ubiquitination modification, USP7 stabilizes Axin protein and suppresses Wnt-induced β-catenin accumulation. Genetic and pharmacological inhibition of USP7 enhances Wnt/β-catenin signaling and modulates Wnt-dependent osteoblast differentiation and adipocyte differentiation.

Axin is a key scaffolding protein of the β-catenin destruction of complex, and cellular response to Wnt is extremely sensitive to the concentration of Axin^52^. Understanding how the concentration of Axin is regulated might help us identify agents to target Wnt/β-catenin signaling. Ubiquitin–proteasome system has been demonstrated as a major system that regulates the concentration of Axin. SIAH mediates Wnt-induced Axin degradation^11^. Tankyrase/RNF146 represents another independent mechanism that promotes Axin degradation; Axin PARsylated by tankyrase is recognized and degraded by RNF146^8,9^. Excessive degradation of Axin in the absence or presence of Wnt stimulation would lead to aberrant activation of Wnt/β-catenin signaling. Therefore, a mechanism likely exists to inhibit excessive degradation of Axin and hyperactivation of Wnt/β-catenin signaling. USP34 was identified as a DUB for Axin, however it controls nuclear localization of Axin and positively regulate Wnt/β-catenin signaling^53^. Our study has identified USP7 as the missing DUB that fulfills this key role. The reason that USP7 has been missed in previous screens is interesting as we did not identify USP7 as a hit in our RNAi Wnt reporter screens^9,11^. It is possible that USP7 is an abundant DUB that cannot be sufficiently knocked down by RNAi. In contrast, expression of USP7 can be eliminated by CRISPR knockout, which exemplifies a key advantage of CRISPR screen over RNAi screen. USP7 is not a significant hit of a gene-trap based Wnt reporter screen in human haploid cell line HAP1^54^. It is likely that HAP1 cells have active p53 signaling^55^, so inactivation of USP7 would inhibit proliferation of HAP1 cells through stabilization of p53. In contrast, the function of p53 is compromised in HEK293T cells.

Our finding of USP7 as a negative regulator of Wnt signaling is not consistent with two previous studies suggesting that USP7 positively regulates Wnt/β-catenin signaling through deubiquitination of β-catenin^23,24^. Although the underlying causes of discrepancies are not clear, one possible explanation is the limitation of early USP7 inhibitors used in the previous studies. Exciting breakthroughs have recently been made in the area of USP7 drug discovery and several potent and selective USP7 inhibitors are reported^29–33^. Although early USP7 inhibitors inhibit Wnt/β-catenin signaling as previously reported^23,24^, new generation of USP7 inhibitors enhance Wnt/β-catenin signaling (Fig. 2g). Importantly, early USP7 inhibitors decrease Wnt reporter at the same concentration at which they inhibit the proliferation of USP7 knockout cells (Fig. 2g), suggesting that their effect on Wnt reporter is likely off-target. In addition, it has been reported that USP7 promotes deubiquitination of β-catenin through directly binding to a region in proximity to the phospho-degron of β-catenin^24^. However, we have failed to detect a clear interaction between USP7 and β-catenin (Fig. 4g), which is consistent with the finding that β-catenin serves as a poor substrate of recombinant USP7 (Fig. 3i). Our study suggests that Axin is a more relevant substrate of USP7 in the Wnt/β-catenin pathway.

Our finding that USP7 inhibitor increases Wnt signaling brings up the concern that USP7 inhibitor might increase proliferation of colorectal cancers, since colorectal cancers often have dysregulated Wnt signaling. To test this possibility, we examined the effect of USP7 inhibitor Almac4 on a panel of seven colorectal cancer cell lines with wild-type or mutated Wnt signaling pathway in colony formation assay. Almac4 inhibited proliferation of RKO (wild-type APC, wild-type β-catenin), HCT116 (β-catenin mutant), and Ls174t (β-catenin mutant) without inhibiting β-catenin signaling in these cells (Supplementary Fig. 6). Interestingly all three cell lines have wild-type p53, suggesting that Almac4 inhibits their proliferation through p53 stabilization. Almac4 did not affect proliferation and β-catenin signaling in the rest of colorectal cancer lines, which have mutant p53 and mutant APC. Interestingly, Almac4 did not have an obvious effect on expression of AXIN1 protein in these cell lines. It is possible these lines express minimal Wnt ligands because of downstream pathway mutation, so Wnt-dependent Axin degradation would be minimal. Mutation of components of β-catenin destruction complex might affect how Axin degradation is regulated. There might also be redundant Dubs that promote Axin deubiquination in colorectal cancer cells. It should be noted that, although we failed to detect any growth promoting activity of USP7 inhibitor, detailed studies are needed to further examine the potential caveat of targeting USP7 in tumors with dysregulated Wnt signaling in the future.

USP7 has a diverse set of substrates and plays important roles in many biological processes. It is possible that USP7 can coordinately regulate several substrates to modulate a biological process. For example, USP7 stabilizes Foxp3 to promote the development and function of regulatory T (Treg) cells^17^, and knockout of USP7 in Treg cells induces lethal systemic autoimmunity in mice^56^. Interestingly, Wnt/β-catenin signaling inhibits transcriptional activity of Foxp3^57^, and expression of stabilized β-catenin in Treg cells induces lethal autoimmunity^58^. Considering the strong negative effect of USP7 on Wnt/β-catenin signaling, it is conceivable that USP7 promotes the function of Treg cells not only through stabilizing Foxp3, but also through stabilizing Axin and blocking Wnt/β-catenin signaling.

It is still not clear whether USP7-mediated deubiquitination serves as a general buffer system to dampen Axin degradation, or it is regulated in a more dynamic fashion. Wnt-induced Axin ubiquitination is prolonged in USP7 knockout cells (Fig. 3j), suggesting that USP7 functions as a mechanism to limit Wnt-induced degradation of Axin. Interestingly, USP7 substrates often have multiple USP7 binding sites. For example, there are two USP7 binding sites in p53^43^, four in MDM2^43,46,59^, two in HDMX^46^, four in Gli^18^. We have identified three binding sites of USP7 in Axin1, two N-terminal to RGS domain and one between RGS domain and GSK3-binding domain. It is possible that multiple interaction sites can enhance USP7 interaction or confer more dynamic regulation. Of note, ^72^PEGS^75^ of a.a. 63–87, which corresponds to the consensus USP7 binding sequence, falls into the second tankyrase binding motif of Axin1^60^. Whether and how USP7-mediated Axin deubiquitination is regulated should be addressed in future studies. In addition, although our data suggest Axin as a major target of USP7 for Wnt regulation, it is possible that regulation of other proteins also contributes to the inhibitory activity of USP7 in Wnt signaling.

Although many small molecule Wnt inhibitors have been identified, few small molecule sensitizers of Wnt/β-catenin signaling exist. USP7 strongly inhibits Wnt/β-catenin signaling through deubiquitination and stabilization of Axin. To our knowledge, USP7 inhibitors represent the most potent agents sensitizing cells to β-catenin signaling beyond GSK3 inhibitors. Selective USP7 inhibitors can potentially be tested in certain disease indications where activation of Wnt signaling is beneficial.

## Methods

### Cell culture and plasmids

HEK293T, Huh7, RKO, U2OS, 3T3-L1, C3H10T1/2 clone 8, HCT116, Ls174t, SW480, HT-29, DLD1, and Caco-2 were purchased from American Type Culture Collection (ATCC), YAPC and ST2 were obtained from DSMZ. HEK293T, YAPC, Huh7, SW480 and 3T3-L1 were grown in DMEM (Cat#11995-040, Invitrogen) with 10% FBS (Cat#1500-500, Seradigm) and penicillin/streptomycin (Cat#10378016, Invitrogen). ST2 and DLD1 were maintained in RPMI-1640 (Cat#22400-071, Invitrogen) with 10% FBS and penicillin/streptomycin. U2OS, HT-29 and HCT116 were grown in McCoy’s 5A (Cat#16600-082, Invitrogen) with 10% FBS and penicillin/streptomycin. RKO, Caco-2, Ls174t and C3H10T1/2 were maintained in MEM (Cat#11095-080, Invitrogen) with 10% FBS and penicillin/streptomycin. All cultures were grown at 37 °C in a 5% CO2 incubator. Cell lines were tested to be free of mycoplasma contamination, and were not subjected to extra authentication steps.

Plasmids were generated using standard recombinant DNA techniques. Site-specific mutagenesis was performed using Q5 Site-Directed Mutagenesis Kit (New England Biolabs). Primers used for cloning are shown in Supplementary Table 1.

### Cell line generation

For CRISPR knockout, cells stably expressing Cas9 were infected with lentivirus expressing indicated gRNA and selected by antibiotics. Unless specifically indicated, pool of knockout cells were used for experiments to avoid clonal variation. All gRNA targeting sequences are listed below: Ctrl (5′ GACCGGAACGATCTCGCGTA3′), *USP7*-g1 (5′CTACGTCGGCTTAAAGAATC3′), *USP7*-g2 (5′ GATTTCGCACAAAACACGGA3′), *CTNNB1*-g1 (5′ACAAAACTGCTAAATGACG3′), *SIAH1* (5′ TAATGCTGTAGCAGTCTGA3′), *AXIN1* (5′ TGAGAAAACCTCTGGTCGTGT3′), *AXIN2* (5′ ATACCCTTTTCTCTCTCCCCAC3′), mouse *Usp7*-g1 (5′ AGACACCAGTTGGCGCTCCG3′), and mouse *Usp7*-g2 (5′ GGCAGATTTCGCACAAAACA3′).

### Genome-wide CRISPR screen and data analysis

We designed five gRNAs against each gene using Illumina Human BodyMap 2.0 and NCBI CCDS data sets. The gRNA library containing 90,000 gRNAs was synthesized using array synthesis and cloned into a lentivirus vector by Cellecta Inc. STF-GFP reporter cells were transduced at MOI 0.5. Fourteen days post virus transduction, cells were treated with 5% Wnt3a CM overnight, and GFP-high and GFP-low cells were sorted using BD FACSAria Cell Sorter. Genomic DNA was collected and subjected to Illumina DNA sequencing for barcode counts. Raw sequencing reads were aligned to the appropriate library using Bowtie, allowing for no mismatches, and counts were generated. The R software package DESeq2 was used to evaluate differential gRNA representation in the form of log2 fold change and *P*-value between the GFP-high and the GFP-low samples. A robust z-score for each gRNA was calculated using the median and mean-absolute deviation across the log2 fold changes. To summarize the results at the gene level, the gRNAs are ranked by the robust z-score, and the statistical significances for each gene enriched toward higher rank (RSA up) were evaluated using the Redundant siRNA Activity (RSA) algorithm. The RSA *P*-value is shown along with the log2 fold change of the five5 gRNAs per gene for the indicated set of genes.

### Flow cytometry assay

Cells were harvested using cell dissociation buffer (Cat#13151014, Invitrogen) and resuspended in FACS buffer (PBS, 1% BSA), then subjected to Cytoflex cytometer (Beckman Coulter Life Sciences). FITC channel was used to collect GFP signal, and raw data was analyzed by FlowJo version 10.

### Quantitative RT-PCR

Total RNA was extracted by using RNeasy Plus mini kit (Cat#74134, QIAGEN) and reverse transcribed by TaqMan reverse transcription reagents (Cat#N8080234, Life Technologies) according to the manufacturer’s instructions. Gene expression levels were detected by TaqMan probes, and all the experiments were performed in quadruplicate. The comparative cycle threshold method was employed to analysis the gene expression with housekeeping gene *GUSB* as an internal normalization control. The Taqman probes used in this study were obtained from Life Technologies, *GUSB* (Hs00939627_m1), *AXIN1* (Hs00394718_m1), *AXIN2* (Hs00610344_m1), *LEF1* (Hs01547250_m1), *CTNNB1* (Hs00355045_m1), *RNF146* (Hs00258475_s1), *Gusb* (Mm01197698_m1), *Axin2* (Mm00443610_m1), *Lef1* (Mm00550265_m1), *Alpl* (Mm00475834_m1), *Pparg* (Mm00440940_m1), *Fap4* (Mm00445878_m1), and *Lpl* (Mm00434764_m1).

### Immunoblotting and immunoprecipitation

For immunoblotting, total cell lysates were prepared by directly lysing cells with NP40 lysis buffer (50 mM Tris-HCl pH 7.5, 150 mM NaCl, 1% Nonidet P-40, 10% Glycerol, 1 mM EDTA, protease inhibitors and phosphatase inhibitors), followed by centrifugation at 15,000*×g* for 15 min at 4 °C. Equal amount of proteins were resolved by SDS-PAGE, transferred to nitrocellulose membrane and probed with indicated antibodies.

The antibodies used are as follows: β-catenin (1:10000, Cat#610154, BD Biosciences), Tubulin (1:100000, Cat#T6074, MilliporeSigma), APC (1:200, Cat#OP44, MilliporeSigma), USP7 (1:1000, Cat#4833S, Cell Signaling Technology), active β-catenin (1:2000, Cat#8814S, Cell Signaling Technology), HA (1:2000, Cat#11867431001, MilliporeSigma), Flag (1:2000, Cat#14793S, Cell Signaling Technology), Myc-tag (1:2000, Cat#2278S, Cell Signaling Technology), GFP (1:2000, Cat#2956S, Cell Signaling Technology), Axin1 (1:1000, Cat#2087 S, Cell Signaling Technology), GSK3β (1:2000, Cat#12456S, Cell Signaling Technology), CK1 (1:1000, Cat#2655S, Cell Signaling Technology),and GST (1:2000, Cat#2625S, Cell Signaling Technology).

Immunoblots were quantified using the ImageJ program (https://imagej.nih.gov/ij/), which measures the integrated density of bands corrected for background. The intensity of target protein and Tubulin bands were quantified by ImageJ, and quantification of normalized band intensity was listed right below the target protein. Uncropped version of immunoblots are shown in Supplementary Figures and Source data file.

For immunoprecipitation experiments, equal amounts of cleared cell lysates were adjusted to the same volume, incubated with normal rabbit IgG control (1:100, Cat#3900S, Cell Signaling Technology) or USP7 primary antibody (1:100, Cat#GTX125894, GeneTex) overnight, and then incubated with magnetic Protein A beads (Cat#88846, Thermo Fisher Scientific) for 2 h at 4 °C. For Myc immunoprecipitation, cell lysates were directly incubated with Anti-Myc tag magnetic beads (Cat#88843, Thermo Fisher Scientific, 20 μl 50% slurry beads to 1 mg total protein.) for 2 h at 4 °C. Then Beads were washed three times with lysis buffer, and bound proteins were eluted in SDS sample buffer for immunoblotting analysis.

### Cytosolic fractionation

Cells were harvested in cold PBS, the cell pellet was resuspended in hypertonic buffer (10 mM Tris-HCl pH 7.5 and 10 mM KCl), and followed by freeze-thaw for four times. Cleared supernatants were collected after full speed centrifugation. Equal amount of proteins were resolved by SDS-PAGE and probed with β-catenin or active β-catenin antibody.

### Transfection

Plasmid transfection was done using FuGene6 (Promega) or Lipofectamine 2000 (Thermo Fisher Scientific) and siRNA transfection was done using Lipofectamine RNAiMAX (Thermo Fisher Scientific) according to manufacturer’s instructions. Lentiviruses were produced using HEK293T cells following the standard virus packaging protocol, and used to generate stable cell lines.

### GST pull-down assay

In vitro purified GST, GST-TRAF, GST-TRAF W165A or GST-TUD was incubated with Flag-Axin1 cell lysate with 20 μl of GSH sepharose 4B resin (Cat#17-0756-01, GE Healthcare) for 2hrs at 4 °C in binding buffer (50 mM Tris-HCl pH 7.4, 150 mM NaCl, 1% Nonidet P-40, 10% Glycerol, 1 mM EDTA and 2 mM DTT, protease inhibitors and phosphatase inhibitors). The beads were then washed with binding buffer three times.

### Ni-NTA pull-down assay

The same amount of purified His-SUMO tag proteins were incubated with purified GST-TRAF or GST-TRAF W165A with 20 μl Ni-NTA resin (Cat#30230, QIAGEN) for 1 hr at 4 °C in binding buffer (50 mM Tris-HCl pH 7.4, 150 mM NaCl, 0.5% Nonidet P-40, 10% Glycerol, 2 mM DTT and 20 mM imidazole). The beads were then washed with binding buffer three times. The proteins were resolved on SDS-PAGE and examined by coomassie staining.

### Deubiquitination assay

For in vivo ubiquitination assay, HEK293T cells were transfected with plasmids encoding HA-tagged ubiquitin, Myc-tagged Axin1 with control vector, wild-type USP7 or C223A mutant. Two days post transfection, cells were treated with 20 μM MG132 for 6hrs and lysed in 100 μl denature lysis buffer (50 mM Tris-HCl pH 7.4, 150 mM NaCl, 1% NP40 and 1% SDS with protease and phosphatase inhibitors). Cell lysates were boiled for 10 min, diluted 10 times in lysis buffer without SDS, and subjected to immunoprecipitation using HA antibody-conjugated beads. Immunoprecipitates were resolved by SDS-PAGE.

For in vitro debuiquitination assay, HEK293T cells co-expressing HA-Ub and Flag-tagged Axin1 or Flag-tagged β-catenin were treated with 10 μM MG132 overnight. Polyubiquitinated Axin1 or β-catenin was purified using Flag-M2 antibody-conjugated beads, then the same amount of beads were incubated with or without 10 ng/µl recombinant full length USP7 (Cat# DB502-25µg, LifeSensors) in the deubiquitination reaction buffer (50 mM Tris-HCl pH 7.4, 150 mM NaCl, 5 mM MgCl2 and 10 mM DTT) at 37 °C for 1 h. The reaction was terminated by the addition of SDS sample buffer. The poly-ubiquitination status of Axin1 and β-catenin was analyzed by HA blotting.

### TUBE assay

In vivo poly-ubiquitination of AXIN1 was evaluated by the TUBE assay. The poly-ubiquitination of target protein was enriched by 20 μM of MG132 treatment. Briefly, equal amount of cell lysates were incubated with agarose-TUBE beads (Cat#UM402, LifeSensors) for 2 h at 4 °C, then the beads were washed three times with lysis buffer, and bound proteins were eluted in SDS sample buffer for immunoblotting analysis.

### STF reporter assay and cell viability assay

HEK293T cells stably expressing the STF-Luc reporter were pretreated with increasing amounts of USP7 inhibitors, following by overnight Wnt3a CM stimulation. The STF-reporter activity was examined by Bright-Glo assay (Promega). Both HEK293T and HEK293T USP7 KO cells were treated with increasing amounts of USP7 inhibitors and viability of cells was assessed after 48 hrs by using CTG assay (Promega).

### Small molecule inhibitors

P5091, MG132 and Bortezomib were purchased from SelleckChem. P22077 and HBX41108 were obtained from TOCRIS. MLN7243 was purchased from Chemietek. Bafilomycin A1 was obtained from MilliporeSigma. The rest of known USP7 inhibitors, WNT974, and TNKS656 were synthesized according to published literature procedures by Novartis.

### Osteoblast differentiation

Briefly, C3H10T1/2 and ST2 cells were plated in normal culture medium (MEM and 10% FBS for C3H10T1/2 and RPMI1640 and 10% FBS for ST2 cells), and allowed to adhere overnight. On the following day, culture medium was replaced with osteoblast differentiation medium (culture medium with 50 µg/ml ascorbic acid, 10 mM β-glycerol phosphate). The medium was changed every other day for 12 days.

To determine alkaline phosphatase enzymatic activity, cells were fixed for 10 min with 10% formalin in PBS and incubated with NBT/BCIP substrate solution (Cat#34042, Thermo Fisher Scientific) for 30 min.

### Adipocyte differentiation

The 3T3-L1 cells were cultured in DMEM with 10% FBS. Adipocyte differentiation was induced post cells growing to confluency by replacing the culture medium containing 1 µM Dexamethasone, 500 µM IBMX, 1 µg/ml insulin for 2 days. On day 3, medium was changed to the culture medium supplemented with insulin (1 µg/ml) and left for 7 days (medium was changed every other day). The lipid droplets were analyzed by Oil-Red O staining (Cat#O1391-250ml, MilliporeSigma).

### Colony formation assay

Colorectal cancer cells were seeded in six-well plates at low density (2,000~5,000 cells per well) and allowed to attach for overnight. DMSO or Amac4 was added directly into each well at indicated concentration. The medium was refreshed every three days with Almac4 at indicated concentration. The plates were washed with PBS and stained with crystal violet when DMSO-treated wells reached confluency.

### Reporting summary

Further information on research design is available in the Nature Research Reporting Summary linked to this article.

## Supplementary information

Supplementary Information

Supplementary dataset

Reporting Summary

Source Data

## Data Availability

Correspondence and requests for materials related to this study should be sent to feng.cong@novartis.com. All data supporting the findings of this study are available within the paper and its Supplementary information files. Raw data and original gel images of all figures are included in the Source Data file. All relevant data are available from the authors on reasonable request.
