# Supplementary material for: USP7 inhibits Wnt/β-catenin signaling through promoting stabilization of Axin
Source: Nat Commun. 2019 Sep 13;10:4184. doi: 10.1038/s41467-019-12143-3 (PMC6744515; doi:10.1038/s41467-019-12143-3)
Supplement: Supplementary file 1 — Supplementary Information [file 41467_2019_12143_MOESM1_ESM.pdf]

Supplementary Information

USP7 inhibits Wnt/β-catenin signaling through promoting stabilization of Axin

Ji et al.

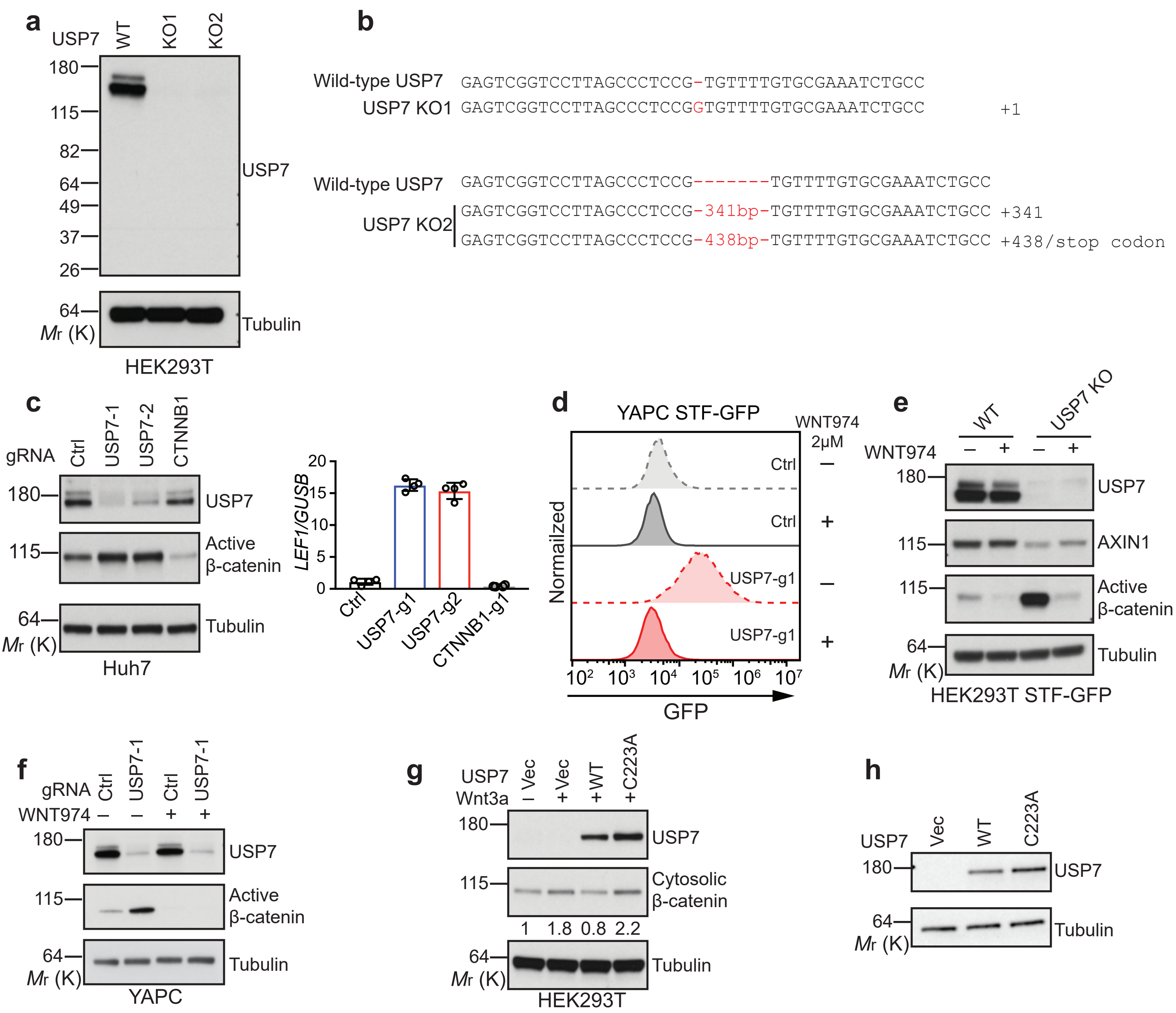

Supplementary Figure 1. Knockout of USP7 increases Wnt/β-catenin signaling. (**a**) Expression of USP7 proteins in parental HEK293T cells and USP7 KO clones. (**b**) Genomic DNA sequencing results depicting mutation of USP7 gene in two individual USP7 KO clones. Two mutant alleles in KO1 are the same. (**c**) Knockout of USP7 enhances accumulation of active β-catenin (left panel) and augments the expression of β-catenin target gene LEF1 in Huh7 cells (right panel). β-catenin knockout serves as an internal control. Error bars denote the SD between four replicates. (**d**) Porcupine inhibitor (WNT974) rescues the high level of STF-GFP in YAPC USP7 depletion cells. Wilde type and USP7 knockout cells were incubated with DMSO or 2μM WNT974 for five days, and STF-GFP was determined by FACS analysis. (**e** and **f**) Porcupine inhibitor WNT974 represses the accumulation of active β-catenin in USP7 knockout HEK293T (**e**) and YAPC cells (**f**) in the absence of exogenous Wnt. Wilde-type and USP7 knockout cells were incubated with DMSO or 2μM WNT974 for five days. (**g**) Overexpression of wild-type (WT) USP7, but not the C223A mutant, represses Wnt3a-induced accumulation of cytosolic β-catenin in HEK293T cells. (**h**) Expression of ectopically expressed wild-type USP7 and the C223A mutant in USP7 KO cells. Control for Fig. 1i. Source data is provided as Soruce Data file.

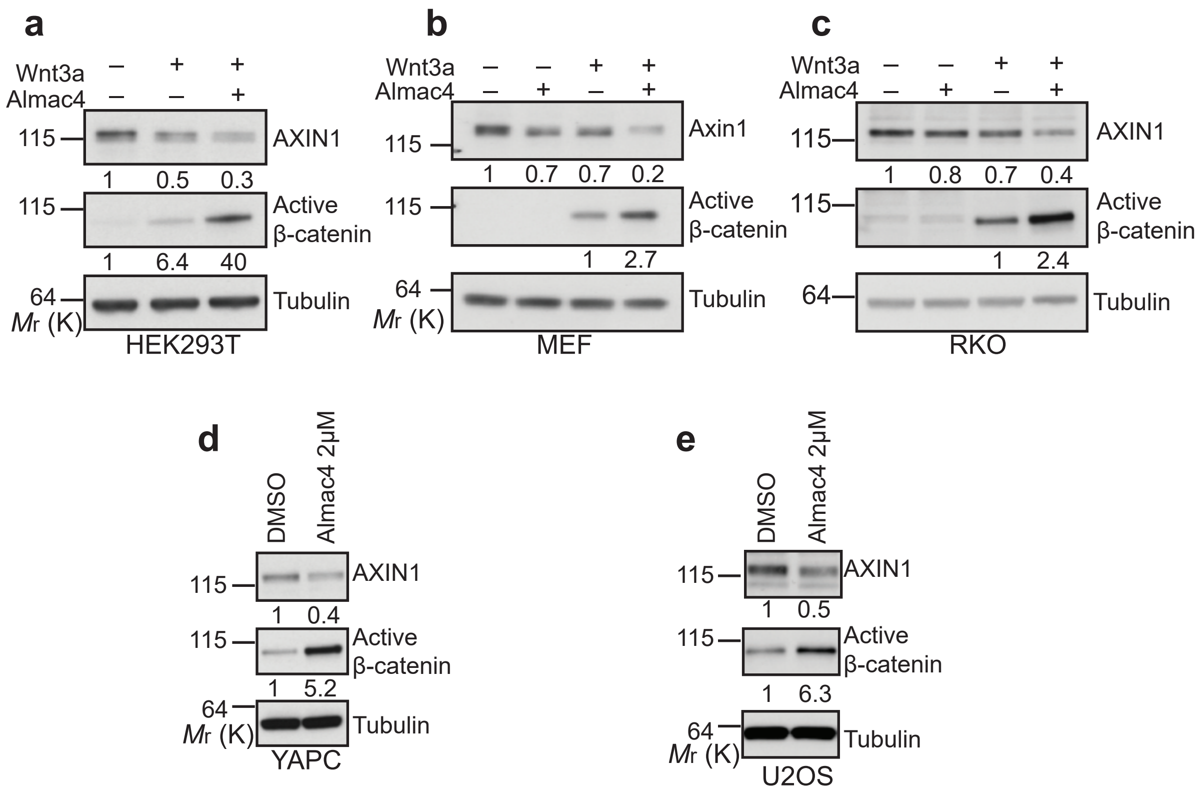

Supplementary Figure 2. USP7 inhibitor Almac4 promotes the Axin degradation and active β-catenin accumulation in multiple cell lines. (**a**) Almac4 promotes the AXIN1 degradation and active β-catenin accumulation in HEK293T. This is the same experiment as shown Fig. 2**c** with the AXIN1 blot included. (**b** and **c**) Almac4 enhances accumulation of active β-catenin and AXIN1 degradation in MEF (**b**) and RKO (**c**) cells. MEF and RKO cells were pretreated with DMSO or 2μM Almac4 for 24hrs and treated with Wnt3a CM overnight. 10% Wnt3a CM for RKO and 2% Wnt3a CM for MEF. (**d**) Almac4 promotes the AXIN1 degradation and active β-catenin accumulation in YAPC. This is the same experiment as shown Fig. 2e with the AXIN1 blotting included. (**e**) Almac4 promotes the AXIN1 degradation and active β-catenin accumulation in U2OS. U2OS cells were treated with DMSO or 2μM Almac4 for 2 days and the active β-catenin and AXIN1 were determined by western blotting. Source data is provided as Soruce Data file.

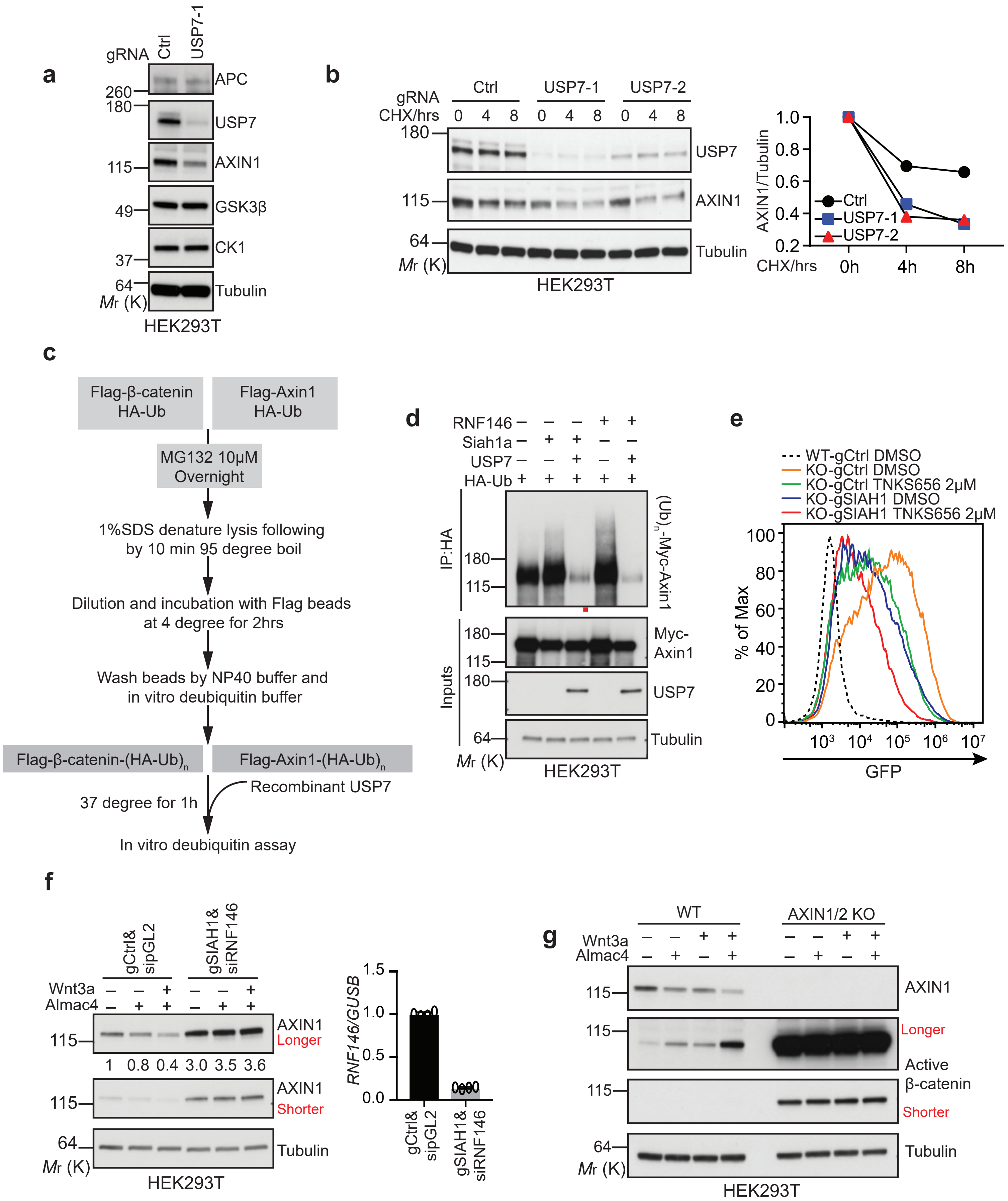
a
gRNA Ctrl USP7-1
APC
260
180
USP7
AXIN1
115
49
GSK3β
CK1
37
64
Tubulin
Mr (K)
HEK293T
b
gRNA Ctrl USP7-1 USP7-2
CHX/hrs 0 4 8 0 4 8 0 4 8
180
USP7
115
AXIN1
64
Tubulin
Mr (K)
HEK293T
AXIN1/Tubulin
1.0
0.8
0.6
0.4
0.2
Ctrl
USP7-1
USP7-2
CHX/hrs 0h 4h 8h
c
Flag-β-catenin HA-Ub
Flag-Axin1 HA-Ub
MG132 10μM Overnight
1%SDS denature lysis following by 10 min 95 degree boil
Dilution and incubation with Flag beads at 4 degree for 2hrs
Wash beads by NP40 buffer and in vitro deubiquitin buffer
Flag-β-catenin-(HA-Ub)n
Flag-Axin1-(HA-Ub)n
Recombinant USP7
37 degree for 1h
In vitro deubiquitin assay
d
RNF146 – – – + +
Siah1a – + + – –
USP7 – – + – +
HA-Ub + + + + +
IP:HA
180
115
(Ub)n-Myc-Axin1
Inputs
180
115
Myc-Axin1
180
USP7
64
Tubulin
Mr (K)
HEK293T
e
WT-gCtrl DMSO
KO-gCtrl DMSO
KO-gCtrl TNKS656 2μM
KO-gSIAH1 DMSO
KO-gSIAH1 TNKS656 2μM
% of Max
100
80
60
40
20
0
10³ 10⁴ 10⁵ 10⁶ 10⁷
GFP
f
gCtrl& sipGL2
gSIAH1& siRNF146
Wnt3a – – + – – +
Almac4 – + + – + +
115
AXIN1
Longer
1 0.8 0.4 3.0 3.5 3.6
115
AXIN1
Shorter
64
Tubulin
Mr (K)
HEK293T
RNF146/GUSB
1.5
1.0
0.5
0.0
gCtrl& sipGL2
gSIAH1& siRNF146
g
WT
AXIN1/2 KO
Wnt3a – – + + – – + +
Almac4 – + – + – + – +
115
AXIN1
115
Longer
Active β-catenin
115
Shorter
64
Tubulin
Mr (K)
HEK293T

Supplementary Figure 3. USP7 regulates Axin stability through counteracting RNF146 and SIAH1. (**a**) USP7 knockout inhibits the protein expression of AXIN1, but not other major components of the β-catenin destruction complex. (**b**) USP7 knockout enhances Wnt-induced Axin degradation. This is an independent repeat of the same experiment shown in Fig. 3e. (**c**) Infographic description of in vitro deubiquitination assay. (**d**) Overexpression of USP7 attenuates both Siah1a and RNF146 overexpression-induced poly-ubiquitination of Axin1. HEK293T cells were transiently transfected with indicated plasmids. Two days post transfection, cells were treated with 10μM bortezomib for 6hrs before harvesting for ubiquitination assay. (**e**) Knock-out of SIAH1, treatment of TNKS inhibitor (TNKS656), or in combination, represses STF-GFP activity in USP7 null HEK293T cells. HEK293T STF-GFP USP7 KO cells bearing control or SIAH1 gRNA were treated with DMSO or 2μM TNKS656 for three days. The STF-GFP reporter activity was determined by FACS assay. (**f**) Deficiency of SIAH1 and RNF146 abolishes Almac4-induced AXIN1 degradation in HEK293T cells. The control and SIAH1 gRNA bearing HEK293T cells were transfected with sipGL2 and siRNF146 respectively. Two days post transfection, cells were treated with DMSO or 1μM Almac4 for 24hrs, and then followed by 10% Wnt3a CM overnight stimulation. The RNF146 knockdown efficiency was determined by RT-PCR. Error bars denote the SD between four replicates. (**g**) Almac4-induced active β-catenin accumulation is abolished in HEK293T AXIN1/AXIN2 double knockout cells. Wild-type and AXIN1/AXIN2 double knockout HEK293T cells were pretreated with DMSO or 1μM Almac4 for 24hrs, then followed by 10% Wnt3a CM overnight stimulation. Source data is provided as Soruce Data file.

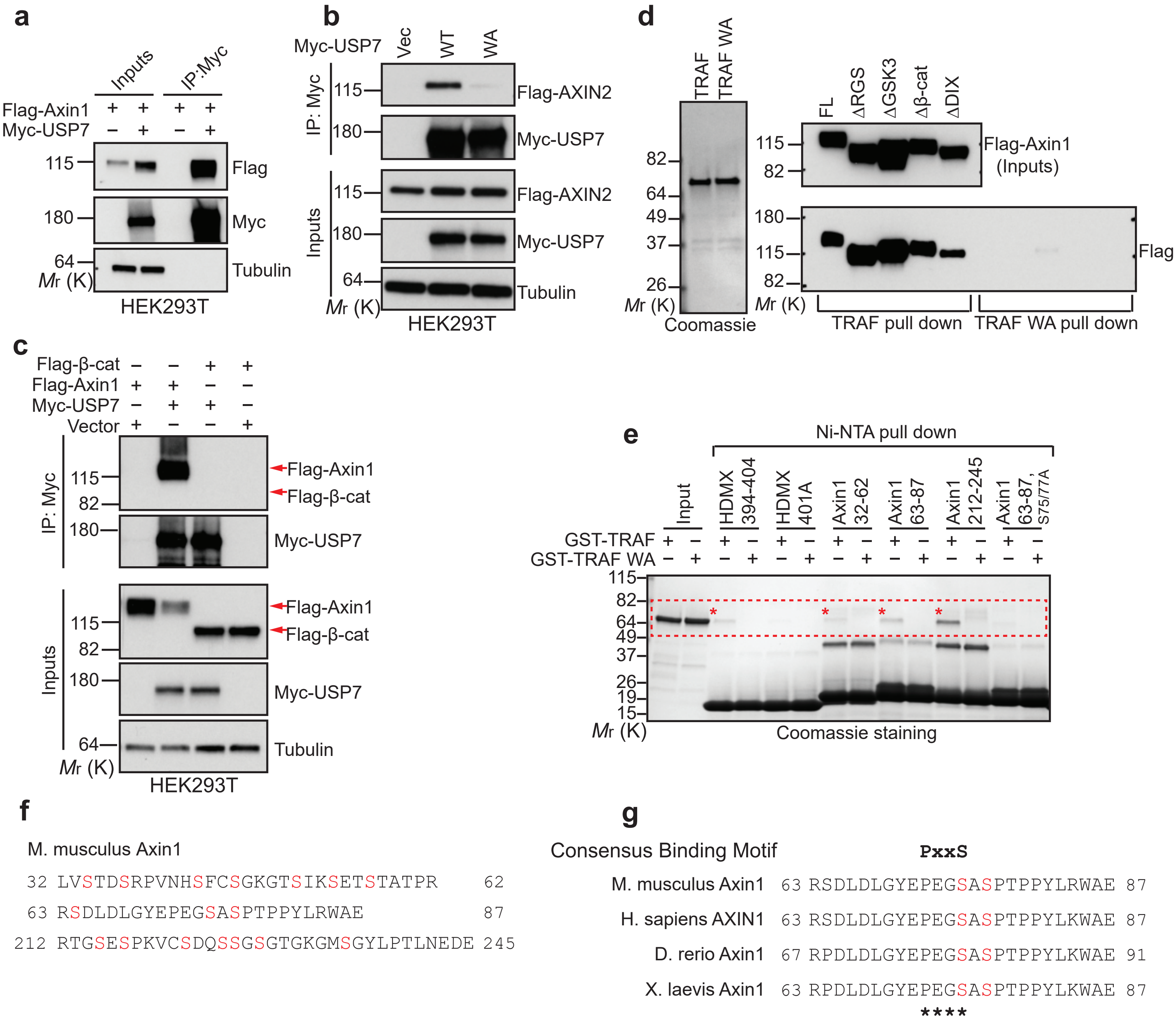

Supplementary Figure 4. Physical interaction between USP7 and Axin. (**a**) Ectopically expressed Axin1 and USP7 interact with each other in the co-immunoprecipitation assay. (**b**) Ectopically expressed Flag-AXIN2 selectively interacts with wild-type USP7, but not W165A mutant, in co-immunoprecipitation assay. (**c**) USP7 has much stronger binding to Axin than β-catenin. HEK293T cells were transiently transfected with indicated plasmids. Two days post transfection, total cell lysates were prepared for immunoprecipitation using anti-Myc antibody. The amount of Flag-Axin1 expression plasmid was one third of Flag-β-catenin expression plasmid in the USP7 co-transfection group. (**d**) RGS domain, β-catenin binding domain, GSK3-binding domain, and DIX domain of Axin1 are not required for the binding between Flag-Axin1 and GST-TRAF in GST-pulldown assay (right panel). Quality of GST fusion proteins was determined by coomassie staining (left panel). Cell lysates expressing full length Flag-tagged Axin1 or its deletion mutants were subjected to GST pulldown assay using GST-TRAF and GST-TRAF W165A.(**e**) Purified GST-TRAF interacts with purified His-SUMO tagged Axin1 a.a. 32-62, a.a.63-87 and a.a. 212-245, but not a.a. 63-87 S75/77A mutant in the Ni-NTA pull down assay. HDMX a.a. 394-404 and its S401A serve as positive and negative control respectively. (**f**) Amino acid sequence of three USP7-binding peptides of Axin1. Ser residues are highlighted in red. (**g**) Alignment of mouse Axin1 a.a. 63-87 with Axin of different species. PxxS motif is a consensus binding motif of USP7 TRAF domain. Source data is provided as Soruce Data file.

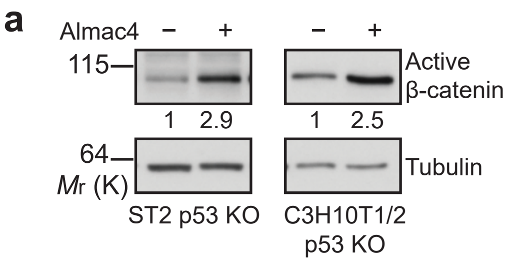

Supplementary Figure 5. Almac4 promotes the accumulation of active β-catenin in ST2, C3H10T1/2 and 3T3-L1 cells. ST2 p53 KO, C3H10T1/2 p53 KO (**a**) and 3T3-L1 (**b**) cells were incubated with DMSO or 2μM Almac4 for two days. The cytosolic fractionation was extracted for active β-catenin immunoblotting. Source data is provided as Soruce Data file.

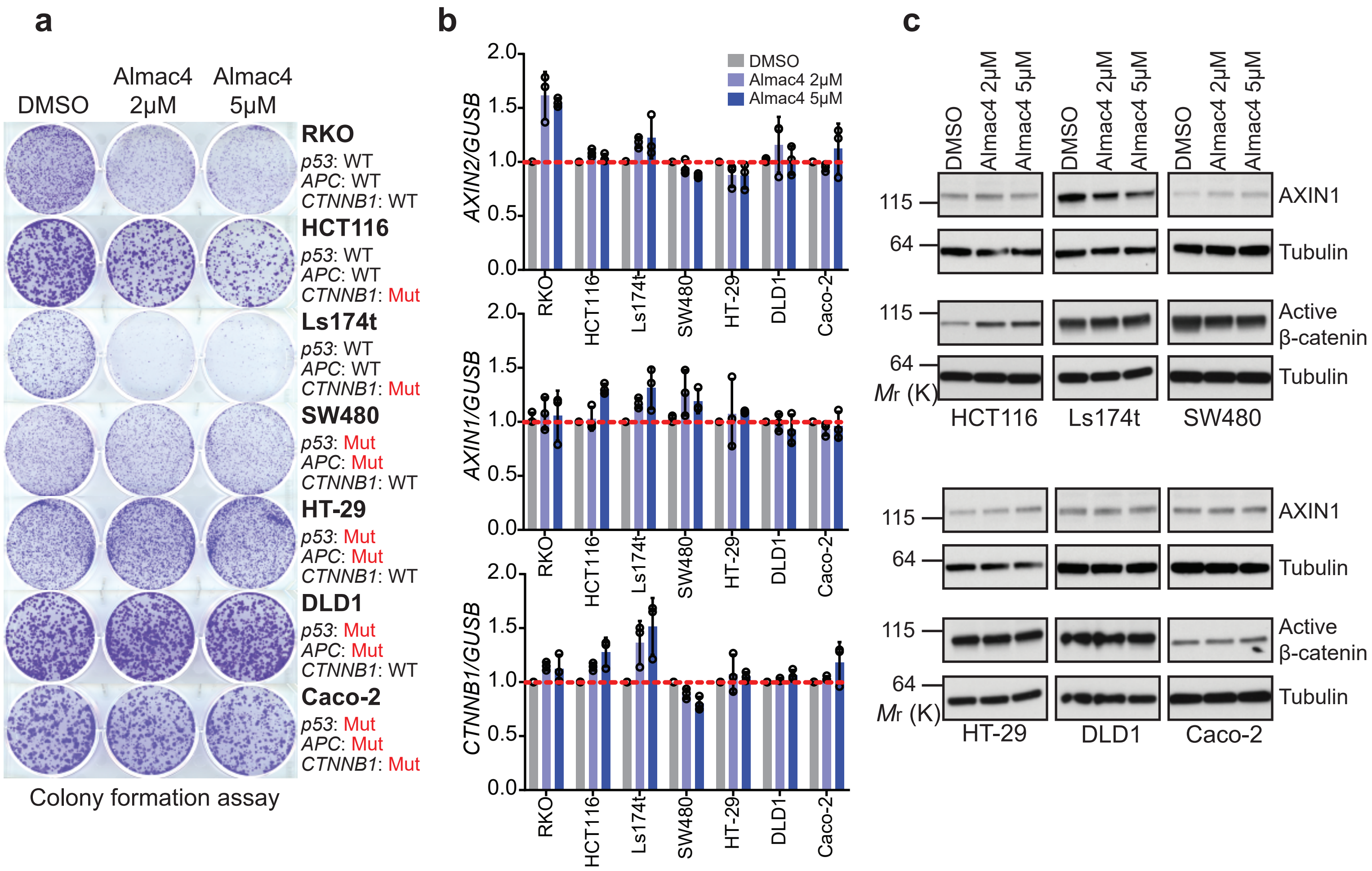

Supplementary Figure 6. Effects of Almac4 on proliferation and Wnt/β-catenin signaling in a panel of colorectal cancer cell lines. (**a**) Effect of Almac4 on proliferation of colorectal cancer lines in colony formation assay. Cells were seeded in 6 well plates and treated with DMSO, 2μM or 5μM Almac4. Drug-containing medium was refreshed every 3 days. Cells were fixed and stained with crystal violet when DMSO control wells reached confluency. (**b** and **c**) Indicated colon cancer lines were incubated with DMSO, 2μM or 5μM Almac4 for two days. mRNA expression of AXIN2, AXIN1 and CTNNB1 was assessed by RT-PCR; Error bars denote the SD between three replicates (**b**), and protein expression of AXIN1 and active β-catenin was determined by immunoblotting (**c**). Source data is provided as Soruce Data file.

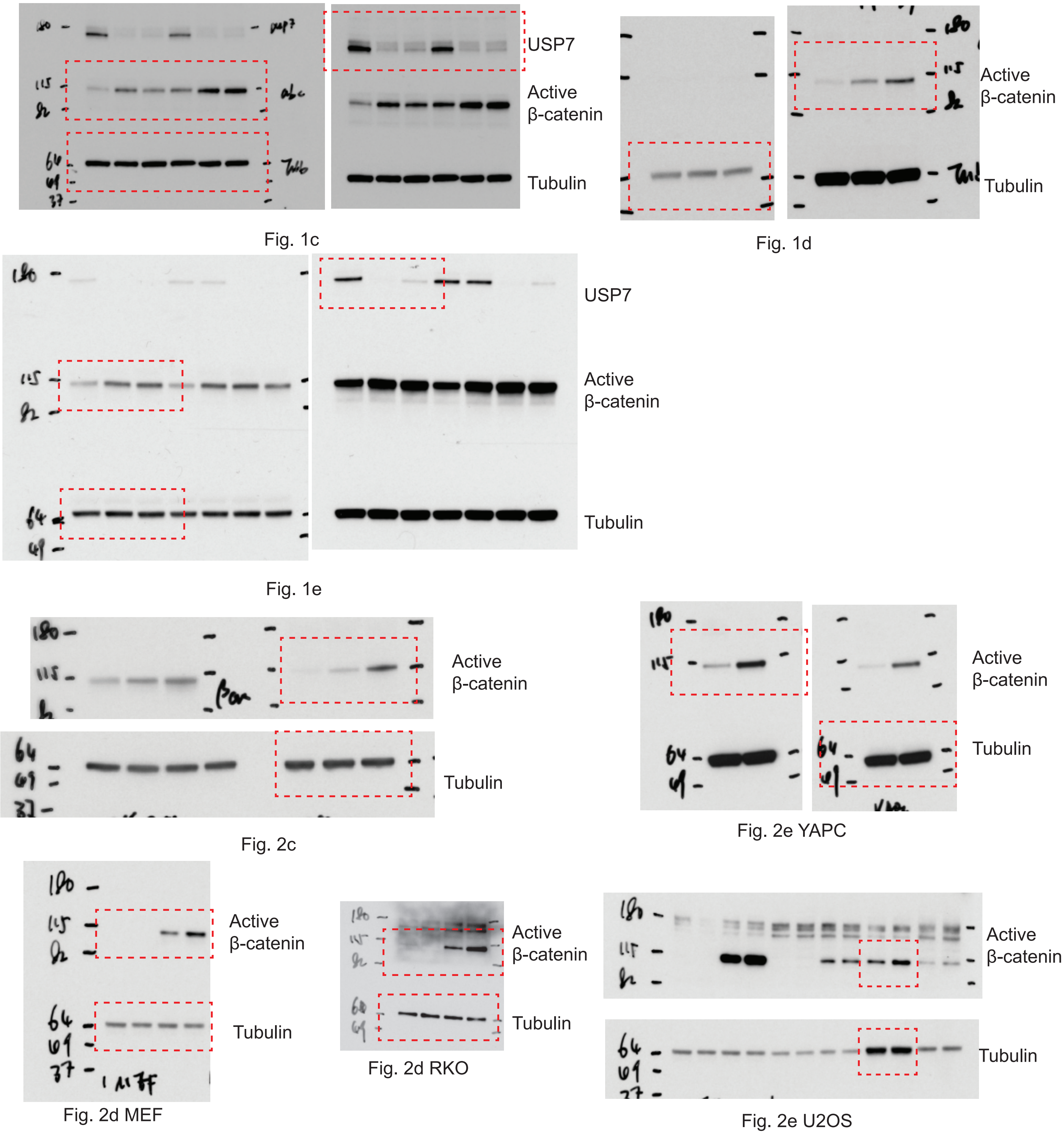

Supplementary Figure 7. Uncropped blots of Figure 1 and 2.

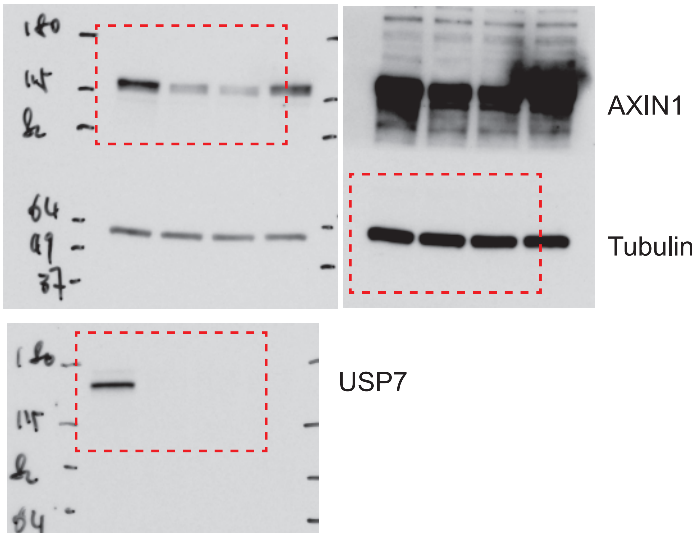

Fig. 3a

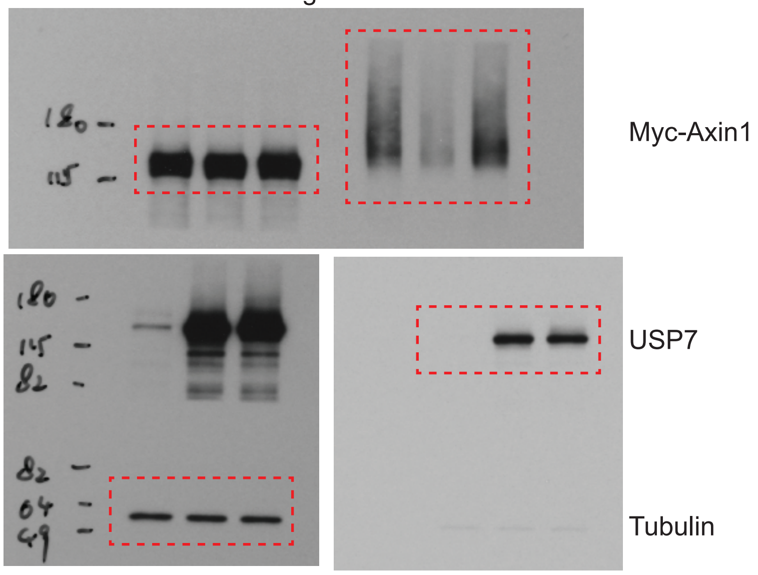

Fig. 3g

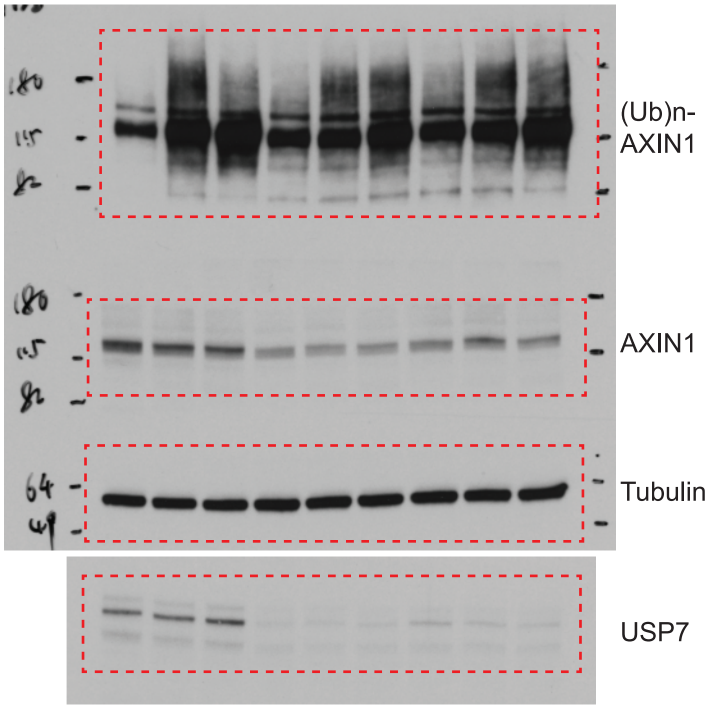

Fig. 3j

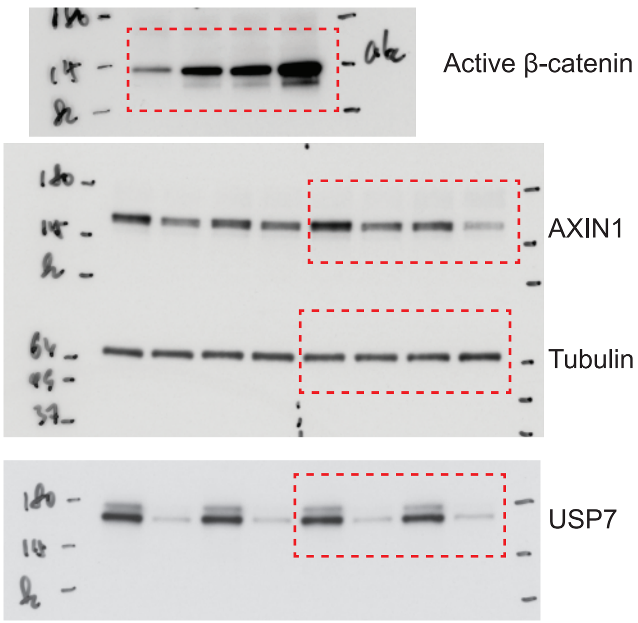

Fig. 3d

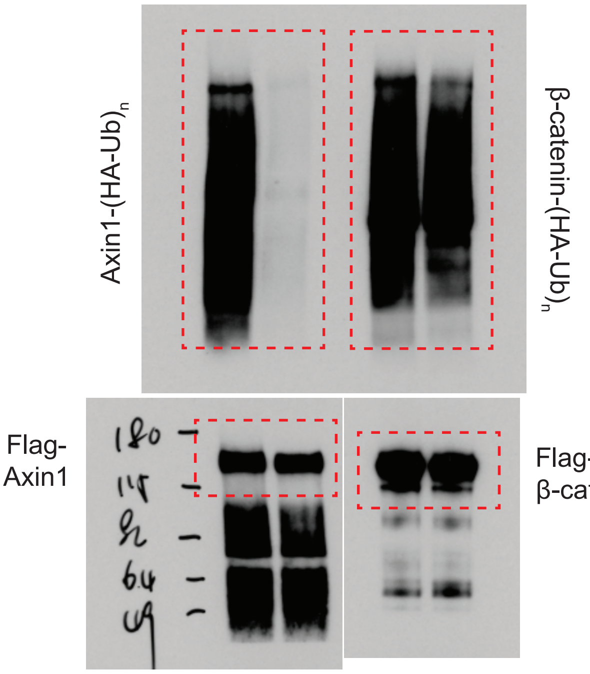

Fig. 3i

Supplementary Figure 8. Uncropped blots of Figure 3.

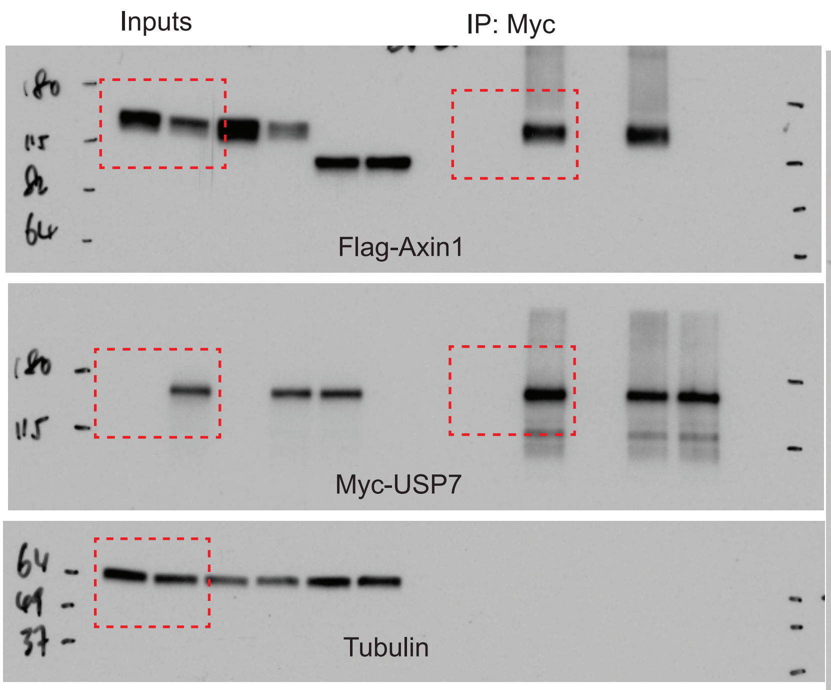

Fig. 4a

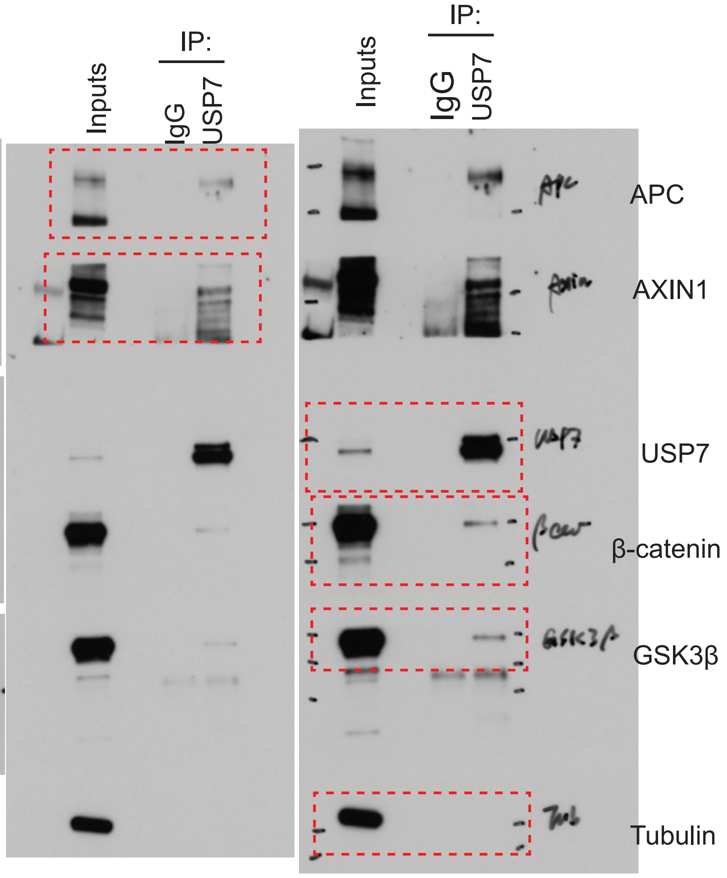

Fig. 4b

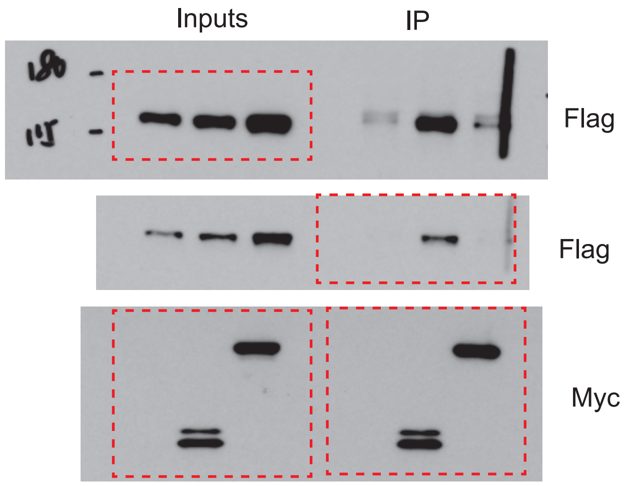

Fig. 4d

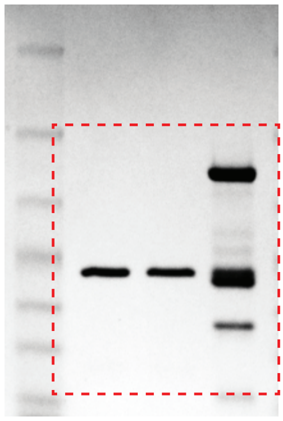

Fig. 4e Coomassie

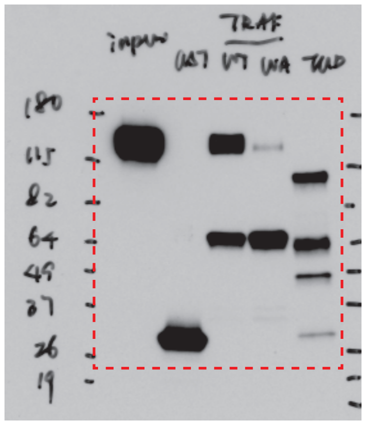

Fig. 4e GST&Flag blotting

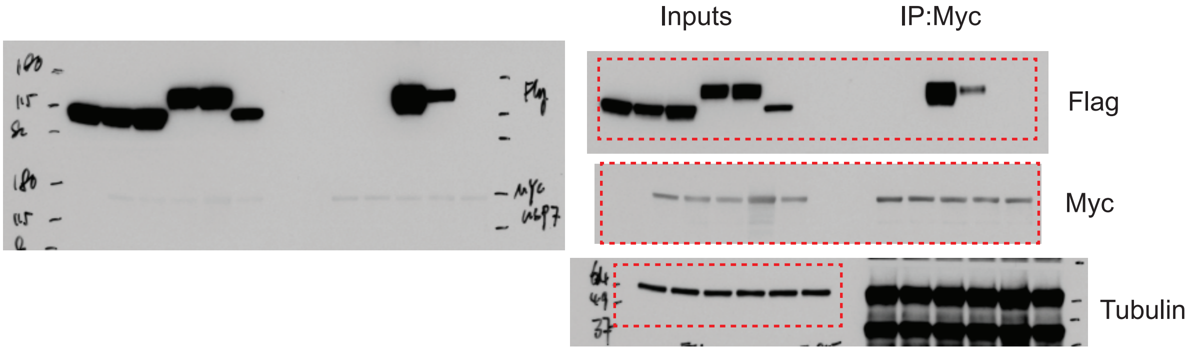

Fig. 4g

Supplementary Figure 9. Uncropped blots of Figure 4.

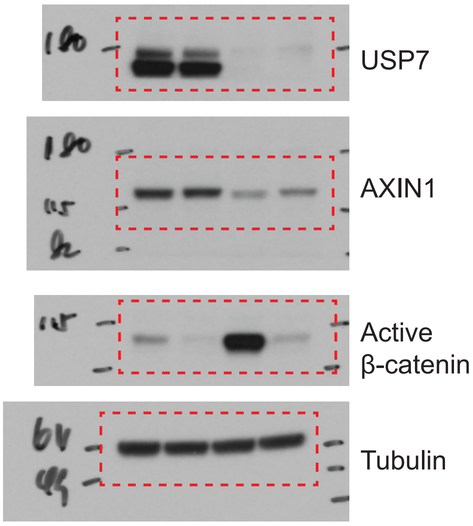

Supplementary Fig. 1e

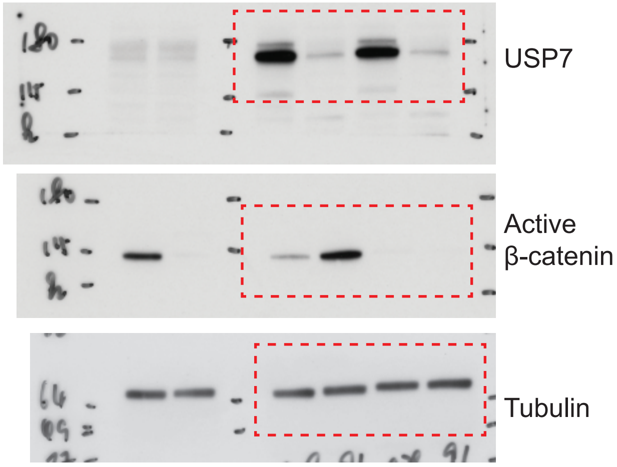

Supplementary Fig. 1f

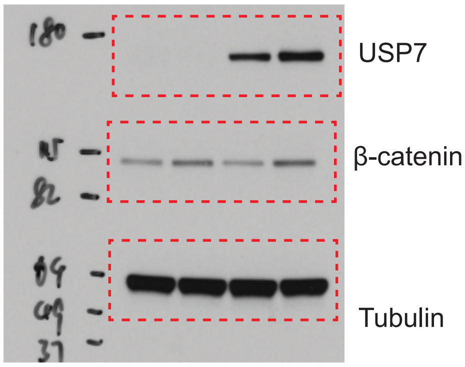

Supplementary Fig. 1g

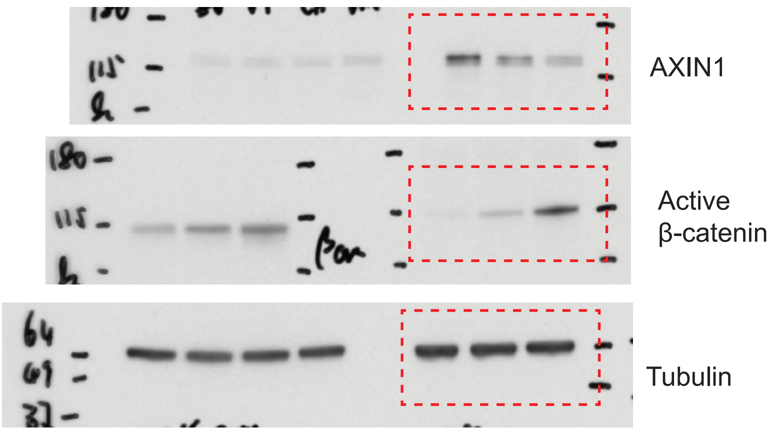

Supplementary Fig. 2a

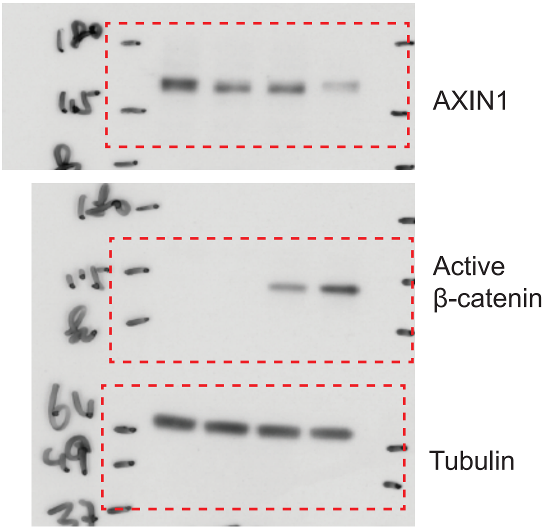

Supplementary Fig. 2b

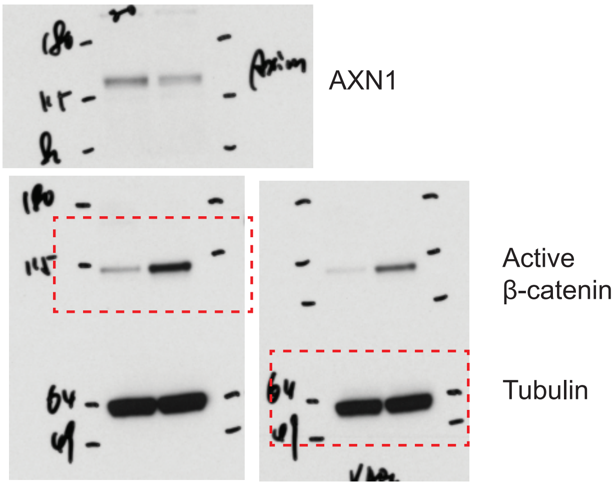

Supplementary Fig. 2d

Supplementary Figure 10. Uncropped blots of Supplementary Figure 1 and 2.

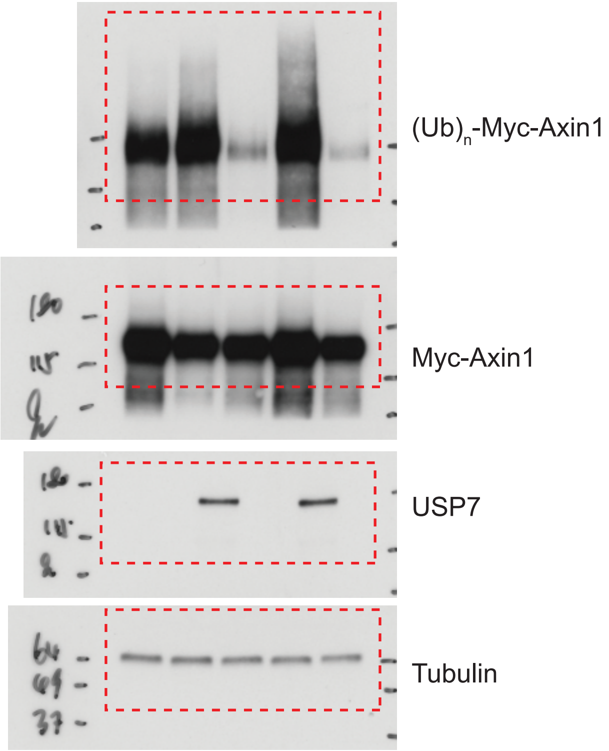

Supplementary Fig. 3d

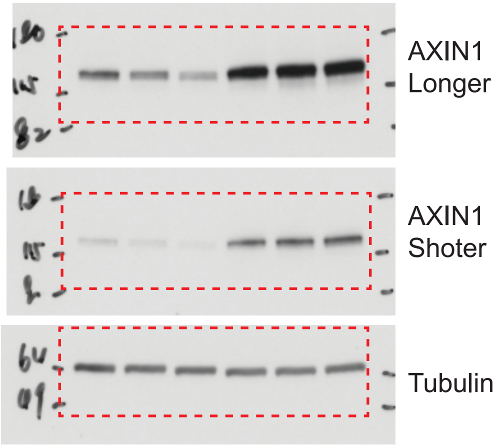

Supplementary Fig. 3f

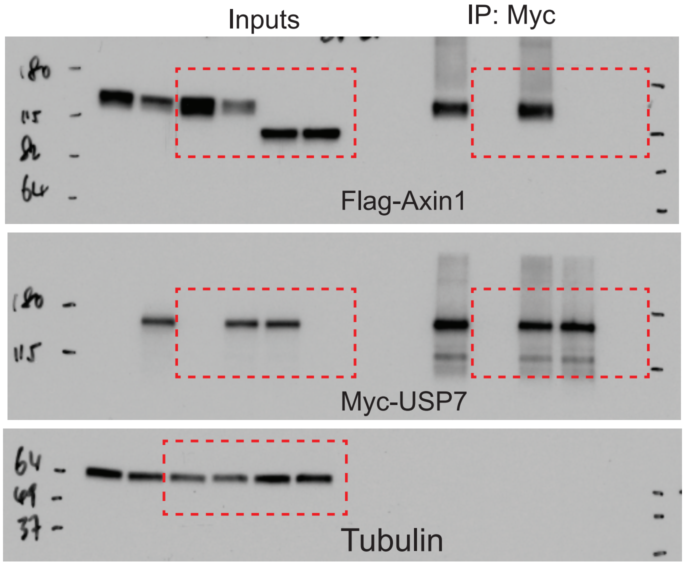

Supplementary Fig. 4c

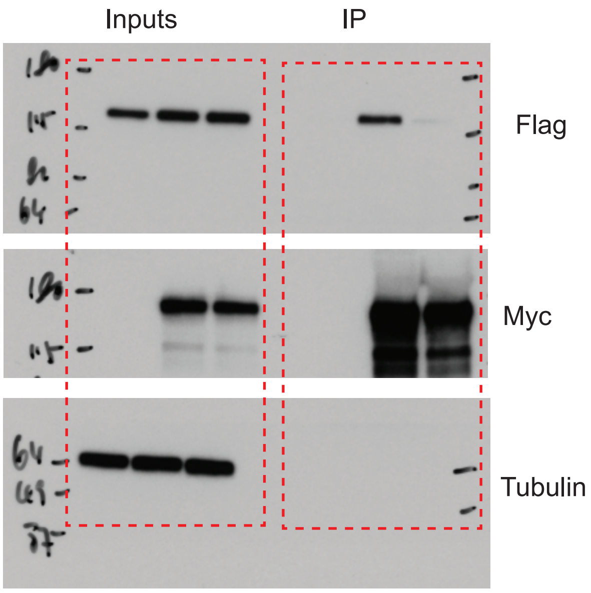

Supplementary Fig. 4b

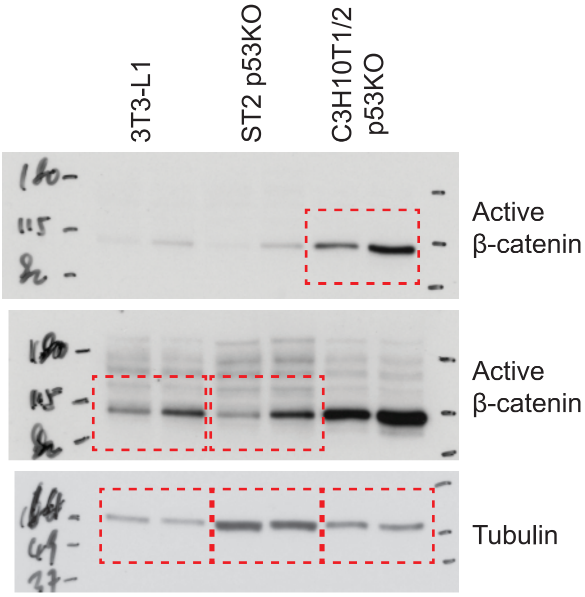

Supplementary Fig. 5a, 5b

Supplementary Figure 11. Uncropped blots of Supplementary Figure. 3, 4 and 5.

Supplementary Table 1. List of primers used in this study

Primer name	Primer sequence (5'-3')	Description
Full length USP7 F	ATGAATCATCAACAGCAACA	Clone full length USP7 gene
Full length USP7 R	CTAGTTGTGGATCTTGATGG	Clone full length USP7 gene
USP7 C223A muation F	AACCAGGGCGCCACAGCCTACATGAACTCCCTGCT	Introduce C223A mutation to USP7
USP7 C223A muation R	AGCAGGGAGTTCATGTAGGCTGTGGCGCCCTGGTT	Introduce C223A mutation to USP7
USP7 W165A muation F	TTCACAAAGAGAACGACGCGGGCTTCAGCAACTTCA	Introduce W165A mutation to USP7
USP7 W165A muation R	TGAAGTTGCTGAAGCCCGCGTCGTTCTCTTTGTGAA	Introduce W165A mutation to USP7
TRAF domain of USP7 F	AATCATCAACAGCAACA	Clone TRAF domain of USP7
TRAF domain of USP7 R	CTAGTCCCAGGCCACGCCATG	Clone TRAF domain of USP7
TUD domain of USP7 F	GAGGCCCACCTGTACATGCAG	Clone TUD domain of USP7
TUD domain of USP7 R	CTAGTTGTGGATCTTGATGG	Clone TUD domain of USP7
Full length mouse Axin1 F	ATGCAGAGTCCCAAAATGAATG	Clone full length mouse Axin1 gene
Full length mouse Axin1 R	TCAGTCCACCTTTTCCACCT	Clone full length mouse Axin1 gene
mouse Axin1 1-501 F	CAGAGTCCCAAAATGAATG	Clone mouse Axin1 1-501 aa
mouse Axin1 1-501 R	AGTCTTAGCCACATGCCCAC	Clone mouse Axin1 1-501 aa
mouse Axin1 502-863 F	GCAGTGCTAGGGGGTACAGC	Clone mouse Axin1 502-863 aa
mouse Axin1 502-863 R	TCAGTCCACCTTTTCCACCT	Clone mouse Axin1 502-863 aa
mouse Axin1 RGS domain F	AGGTGGGCTGAGTCACTG	Clone mouse Axin1 RGS domain
mouse Axin1 RGS domain R	TGTGTACTCCAAATAAATG	Clone mouse Axin1 RGS domain
mouse Axin1 GSK3 binding domain F	ATCCGTAAGCAGCACCG	Clone mouse Axin1 GSK3 binding domain
mouse Axin1 GSK3 binding domain R	GGGGCCAGAAGGCATTTC	Clone mouse Axin1 GSK3 binding domain
mouse Axin1 β-catenin binding domain F	ATGGCAAGTCACAAGCTG	Clone mouse Axin1 β-catenin binding domain
mouse Axin1 β-catenin binding domain R	TCAAGTCTTAGCCACATGCCC	Clone mouse Axin1 β-catenin binding domain
mouse Axin1 1-31aa F	CAGAGTCCCAAAATGAAT	Clone mouse Axin1 1-31 aa
mouse Axin1 1-31aa R	TTCTCCCTCTTCTCCAGG	Clone mouse Axin1 1-31 aa
mouse Axin1 32-62aa F	CTGGTATCTACTGATTCG	Clone mouse Axin1 32-62 aa
mouse Axin1 32-62aa R	TCTTGGGGTGGCTGTTGAG	Clone mouse Axin1 32-62 aa
mouse Axin1 63-87aa F	CGTTCAGATCTGGATCTG	Clone mouse Axin1 63-87 aa
mouse Axin1 63-87aa R	CTCAGCCCACCTCAAATAT	Clone mouse Axin1 63-87 aa
mouse Axin1 212-245aa F	AGGACAGGCTCAGAGAGT	Clone mouse Axin1 212-245 aa
mouse Axin1 212-245aa R	TTCATCCTCATTCAAAGT	Clone mouse Axin1 212-245 aa
mouse Axin1 246-285aa F	GAATGGAAATGTGACCAAG	Clone mouse Axin1 246-285 aa
mouse Axin1 246-285aa R	CCGTCTACTTGAGGGGGC	Clone mouse Axin1 246-285 aa
mouse Axin1 286-311aa F	TACAACGAAGGCAGAGAG	Clone mouse Axin1 286-311 aa
mouse Axin1 286-311aa R	GGCATAGCCAGAGTTGACG	Clone mouse Axin1 246-285 aa
mouse Axin1 312-347aa F	GGCTATGCCCTGGCCCCAG	Clone mouse Axin1 312-347 aa
mouse Axin1 312-347aa R	CCTGTATGGGGGGATTCCATC	Clone mouse Axin1 312-347 aa
mouse Axin1 445-475aa F	CATTTCCCACCCCGCTAT	Clone mouse Axin1 445-475 aa
mouse Axin1 445-475aa R	TTGCACGTGCTCATCCAG	Clone mouse Axin1 445-475 aa
mouse Axin1 502-535aa F	ACTGCAGTGCTAGGGGGT	Clone mouse Axin1 502-535 aa
mouse Axin1 502-535aa R	GTGGTGGACATGTCTATG	Clone mouse Axin1 502-535 aa
mouse Axin1 536-580aa F	CATGTTCACCATAATTC	Clone mouse Axin1 536-580 aa
mouse Axin1 536-580aa R	GCCTGCGTTCTCGGAAT	Clone mouse Axin1 536-580 aa
mouse Axin1 581-605aa F	ACCACCCTCAGTGCTGG	Clone mouse Axin1 581-605 aa
mouse Axin1 581-605aa R	AGCCTTCTTGGTGTTTCT	Clone mouse Axin1 581-605 aa
mouse Axin1 63-87aa, S75,77A F	CGCCCCCACCCCACCATATTTGAG	Introduce S75A and S77A mutations to mouse Axin1
mouse Axin1 63-87aa, S75,77A R	GCTGCGCCCTCGGGCTCATATCC	Introduce S75A and S77A mutations to mouse Axin1
HMDX 394-404 F	TTCCTGGACCTGGCCCACAGCAGCGAGAGCCAGTGA	Clone human HDMX 394-404 aa
HMDX 394-404 R	TCACTGGCTCTCGCTGCTGTGGGCCAGGTCCAGGAA	Clone human HDMX 394-404 aa
HMDX 394-404 S401A F	TTCCTGGACCTGGCCCACAGCGCCGAGAGCCAGTGA	Clone human HDMX 394-404 aa with S401A mutation
HMDX 394-404 S401A R	TCACTGGCTCTCGGCGCTGTGGGCCAGGTCCAGGAA	Clone human HDMX 394-404 aa with S401A mutation
