# Supplementary material for: USP7 inhibits Wnt/β-catenin signaling through promoting stabilization of Axin
Source: Nat Commun. 2019 Sep 13;10:4184. doi: 10.1038/s41467-019-12143-3 (PMC6744515; doi:10.1038/s41467-019-12143-3)
Supplement: Supplementary file 3 — Reporting Summary [file 41467_2019_12143_MOESM3_ESM.pdf]

nature research

Corresponding author(s): Feng Cong, Ph.D.
feng.cong@novartis.com

Last updated by author(s): May 30, 2019

Reporting Summary

Nature Research wishes to improve the reproducibility of the work that we publish. This form provides structure for consistency and transparency in reporting. For further information on Nature Research policies, see Authors & Referees and the Editorial Policy Checklist.

Statistics

For all statistical analyses, confirm that the following items are present in the figure legend, table legend, main text, or Methods section.

n/a	Confirmed	
☐	☒	The exact sample size (*n*) for each experimental group/condition, given as a discrete number and unit of measurement
☐	☒	A statement on whether measurements were taken from distinct samples or whether the same sample was measured repeatedly
☐	☒	The statistical test(s) used AND whether they are one- or two-sided<br>*Only common tests should be described solely by name; describe more complex techniques in the Methods section.*
☒	☐	A description of all covariates tested
☒	☐	A description of any assumptions or corrections, such as tests of normality and adjustment for multiple comparisons
☐	☒	A full description of the statistical parameters including central tendency (e.g. means) or other basic estimates (e.g. regression coefficient) AND variation (e.g. standard deviation) or associated estimates of uncertainty (e.g. confidence intervals)
☒	☐	For null hypothesis testing, the test statistic (e.g. *F*, *t*, *r*) with confidence intervals, effect sizes, degrees of freedom and *P* value noted<br>*Give P values as exact values whenever suitable.*
☒	☐	For Bayesian analysis, information on the choice of priors and Markov chain Monte Carlo settings
☒	☐	For hierarchical and complex designs, identification of the appropriate level for tests and full reporting of outcomes
☒	☐	Estimates of effect sizes (e.g. Cohen's *d*, Pearson's *r*), indicating how they were calculated

Our web collection on statistics for biologists contains articles on many of the points above.

Software and code

Policy information about availability of computer code

Data collection	Real-time PCR data was collected by software QuantStudio 7 Flex Real-Time PCR System, Thermo Fisher Scientific. FACS data was collected by Cytoflex Flow Cytometer, BECKMAN COULTER Life Sciences. Luciferase data was collected by EnVision, PerkinElmer.
Data analysis	Statistics were calculated by GraphPad Prism. Quantification of western image was done by ImageJ. The FACS data was analyzed by FlowJo V10.

For manuscripts utilizing custom algorithms or software that are central to the research but not yet described in published literature, software must be made available to editors/reviewers. We strongly encourage code deposition in a community repository (e.g. GitHub). See the Nature Research guidelines for submitting code & software for further information.

Data

Policy information about availability of data

All manuscripts must include a data availability statement. This statement should provide the following information, where applicable:

- Accession codes, unique identifiers, or web links for publicly available datasets
- A list of figures that have associated raw data
- A description of any restrictions on data availability

Data supporting the findings of this study are available within the article and its Supplementary Information files. All raw data and original gel images are included in the Source Data file. All relevant data are available from the authors on reasonable request.

Field-specific reporting

Please select the one below that is the best fit for your research. If you are not sure, read the appropriate sections before making your selection.

☒ Life sciences ☐ Behavioural & social sciences ☐ Ecological, evolutionary & environmental sciences

For a reference copy of the document with all sections, see nature.com/documents/nr-reporting-summary-flat.pdf

Life sciences study design

All studies must disclose on these points even when the disclosure is negative.

Sample size	Experiments were performed using sample sizes based on the standard protocols in the field. No statistical test was performed to predetermine sample size
Data exclusions	No data was excluded
Replication	All experiments were repeated at least three times and all attempts to replicate the experiments performed here were successful
Randomization	Sample allocation was random
Blinding	Data acquisition in the studies was conducted in a blinded manner

Reporting for specific materials, systems and methods

We require information from authors about some types of materials, experimental systems and methods used in many studies. Here, indicate whether each material, system or method listed is relevant to your study. If you are not sure if a list item applies to your research, read the appropriate section before selecting a response.

Materials & experimental systems

n/a	Involved in the study	
☐	☒	Antibodies
☐	☒	Eukaryotic cell lines
☒	☐	Palaeontology
☒	☐	Animals and other organisms
☒	☐	Human research participants
☒	☐	Clinical data

Methods

n/a	Involved in the study	
☒	☐	ChIP-seq
☐	☒	Flow cytometry
☒	☐	MRI-based neuroimaging

Antibodies

Antibodies used	β-catenin (1:10000, Cat#610154, BD Biosciences), Tubulin (1:100000, Cat#T6074, MilliporeSigma), APC (1:200, Cat#OP44, MilliporeSigma), USP7 (1:1000, Cat#4833S, Cell Signaling Technology), active β-catenin (1:2000, Cat#8814S, Cell Signaling Technology), HA (1:2000, Cat#11867431001, MilliporeSigma), Flag (1:2000, Cat#14793S, Cell Signaling Technology), Myc-tag (1:2000, Cat#2278S, Cell Signaling Technology), GFP (1:2000, Cat#2956S, Cell Signaling Technology), Axin1 (1:1000, Cat#2087S, Cell Signaling Technology), GSK3β (1:2000, Cat#12456S, Cell Signaling Technology), CK1 (1:1000, Cat#2655S, Cell Signaling Technology),and GST (1:2000, Cat#2625S, Cell Signaling Technology).
Validation	All antibodies used were validated by the respective commercial source for the application used in this study.

Eukaryotic cell lines

Policy information about cell lines

Cell line source(s)	HEK293T, Huh7, RKO, U2OS, 3T3-L1, C3H10T1/2 clone 8, HCT116, Ls174t, SW480, HT-29, DLD1 and Caco-2 were purchased from American Type Culture Collection (ATCC), YAPC and ST2 were obtained from DSMZ.
Authentication	No extra authentication was performed for the cell lines were directly purchased from ATTC and DSMZ
Mycoplasma contamination	All cell lines tested negative for mycoplasma contamination
Commonly misidentified lines (See ICLAC register)	No commonly misidentified lines were used

Flow Cytometry

Plots

Confirm that:

☒ The axis labels state the marker and fluorochrome used (e.g. CD4-FITC).

☒ The axis scales are clearly visible. Include numbers along axes only for bottom left plot of group (a 'group' is an analysis of identical markers).

☐ All plots are contour plots with outliers or pseudocolor plots.

☐ A numerical value for number of cells or percentage (with statistics) is provided.

Methodology

Sample preparation	Cells were harvested using cell dissociation buffer and resuspended in FACS buffer (PBS, 1% BSA), then subjected to Cytoflex cytometer.
Instrument	Cytoflex cytometer (Beckman Coulter Life Sciences)
Software	FlowJo V10
Cell population abundance	A minimum of 10,000 cells per sample were analyzed
Gating strategy	Live cells were gated by FSC-A and SSC-A and singlets were derived from live cell population by FSC-Width and FSC-A. The STF-GFP signal was collected in FTIC channel from singlet population.

☐ Tick this box to confirm that a figure exemplifying the gating strategy is provided in the Supplementary Information.
